# Evaluating DFHBI-Responsive RNA Light-Up Aptamers as Fluorescent Reporters for Gene Expression

**DOI:** 10.1021/acssynbio.3c00599

**Published:** 2023-11-22

**Authors:** Alicia Climent-Catala, Ivan Casas-Rodrigo, Suhasini Iyer, Rodrigo Ledesma-Amaro, Thomas E. Ouldridge

**Affiliations:** † Imperial College Centre for Synthetic Biology, London SW7 2AZ, U.K.; ‡ Department of Chemistry, Imperial College London, London SW7 2AZ, U.K.; § Department of Biosystems Science and Engineering, ETH Zurich, CH-4058 Basel, Switzerland; ∥ Department of Bioengineering, Imperial College London, London SW7 2AZ, U.K.; ⊥ Department of Life Sciences, Imperial College London, London SW7 2AZ, U.K.

**Keywords:** synthetic biology, RNA light-up aptamers, gene expression, E. coli, dynamics, reporters

## Abstract

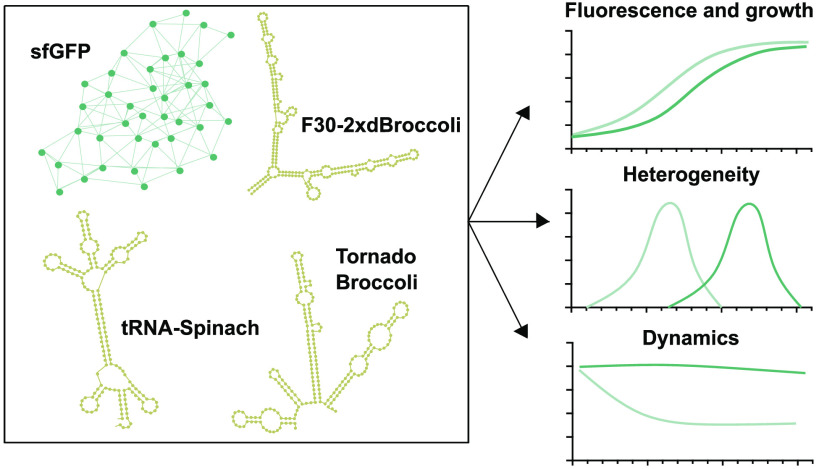

Protein-based fluorescent reporters have been widely used to characterize and localize biological processes in living cells. However, these reporters may have certain drawbacks for some applications, such as transcription-based studies or biological interactions with fast dynamics. In this context, RNA nanotechnology has emerged as a promising alternative, suggesting the use of functional RNA molecules as transcriptional fluorescent reporters. RNA-based aptamers can bind to nonfluorescent small molecules to activate their fluorescence. However, their performance as reporters of gene expression in living cells has not been fully characterized, unlike protein-based reporters. Here, we investigate the performance of three RNA light-up aptamers—F30-2xdBroccoli, tRNA-Spinach, and Tornado Broccoli—as fluorescent reporters for gene expression in *Escherichia coli* and compare them to a protein reporter. We examine the activation range and effect on the cell growth of RNA light-up aptamers in time-course experiments and demonstrate that these aptamers are suitable transcriptional reporters over time. Using flow cytometry, we compare the variability at the single-cell level caused by the RNA fluorescent reporters and protein-based reporters. We found that the expression of RNA light-up aptamers produced higher variability in a population than that of their protein counterpart. Finally, we compare the dynamical behavior of these RNA light-up aptamers and protein-based reporters. We observed that RNA light-up aptamers might offer faster dynamics compared to a fluorescent protein in *E. coli*. The implementation of these transcriptional reporters may facilitate transcription-based studies, gain further insights into transcriptional processes, and expand the implementation of RNA-based circuits in bacterial cells.

## Introduction

Protein-based fluorescent reporters have been essential to the progress and development of molecular and cellular biology. The multicolour fluorescent proteins developed in the Tsien lab^1−5^ have been vital to detect, characterize, and understand a plethora of biological processes such as gene expression and cell dynamics via labeling and tracking proteins in living cells.^6^ However, for some applications, these protein-based reporters may present some drawbacks. For instance, their large size, cost in terms of cellular resources, and their ability to measure activity only at the protein level, providing an indirect measure of the transcription process, can be inconvenient. Also, the slow maturation and deactivation of some fluorescent proteins may affect the detection of rapid processes, for example, in gene expression dynamics.^7^

Recent advances in nucleic acid engineering and RNA biology^8,9^ have enabled the emergence and development of alternative reporters based on RNA molecules.^10−13^ RNA reporters have potential advantages compared to protein-based reporters: the dynamics of activation and degradation are potentially faster, their expression may impose a lower metabolic burden, as it only involves the transcription process, and their structure and function are predictable and versatile due to Watson–Crick base pairing.^14,15^ Therefore, some attention has been devoted to RNA reporters for certain applications such as implementing RNA-based circuits with fluorescent reporters,^16,17^ synthetic circuits requiring fast dynamics, transcription-based studies, or sequence-specific RNA–RNA and RNA–protein interactions.^13^

RNA aptamers are transcripts that have been optimized for high-affinity binding to specific ligands by exponential enrichment (SELEX).^18^ RNA light-up aptamers have been evolved to bind and stabilize specific nonfluorescent small molecules. Once the RNA light-up aptamer binds with their cognate ligand and the aptamer-ligand complex is formed, a fluorescence signal is produced and can easily be detected.^19^ A plethora of light-up aptamers have been described with different properties such as binding affinity for their small ligand, fluorescence wavelengths, and brightness. Among them, we can highlight the Malachite green,^20,21^ Spinach,^22^ Broccoli,^23,24^ Corn,^25^ Beetroot,^26^ Mango,^27,28^ and Pepper light-up aptamers.^29,30^

Over the past few years, these RNA light-up aptamers have been mainly implemented as tags to track and localize mRNA molecules in bacterial and mammalian cells using fluorescence microscopy,^25,27^ as biosensors for therapy and diagnostic applications,^31−33^ and as reporters in synthetic biology for in vitro and in vivo applications.^16,17,34^ However, it remains a challenge to express fluorescent aptamers in living cells due to several factors affecting their performance, such as transport of the fluorophore across the membrane, toxicity to the cell, or stability of the aptamer. Regarding the latter, several strategies have been proposed to improve RNA stability in living cells and prevent degradation of the RNA aptamer. For instance, tRNA scaffolds,^35^ introns, and ribozymes^36−38^ have been proposed as strategies to embed and protect the RNA light-up aptamers in cells.

Here, we explore the performance of RNA light-up aptamers as nonprotein-based reporters for transcription in bacterial cells that remains so far barely characterized. Pothoulakis et al. reported good expression signals from the tRNA-Spinach light-up aptamer in *Escherichia coli*, establishing a proof of concept for this RNA aptamer as a characterization tool to measure transcription and protein production from the same transcript.^34^ In this work, we examine the expression of three DFHBI-responsive RNA light-up aptamers in living cells to study their expression levels, effects on cell growth, expression variability within the sample, and activation and deactivation dynamics to have a deeper understanding of their performance as transcriptional reporters in living cells. We investigate and fully characterize the performance of three DFHBI-responsive RNA light-up aptamers, two of them embedded in scaffolds—F30-2xdBroccoli^23^ and tRNA-Spinach^22,34^—and one in a circular structure—the Tornado expression system with a Broccoli RNA aptamer.^36^ These aptamers mimic the green fluorescent protein upon binding to a specific GFP-like fluorophore (DFHBI-1T). DFHBI-1T shows a brighter fluorescence signal than other versions or fluorophores and a low fluorescent background, which increases the signal-to-noise ratio during fluorescence imaging.^39^ Our results suggest that DFHBI-responsive RNA aptamers are suitable transcriptional reporters to evaluate gene expression in microplate readers for the cell population. These reporters show high signal-to-noise ratios for the fluorescence signal even though the absolute signal is lower than the protein reporter sfGFP.^40^ We also evaluated and characterized the variability of these RNA light-up aptamers at the single-cell level to provide insights into their implementation as fluorescent reporters. Our results show higher variability in gene expression for the transcriptional reporters compared to protein-based reporters. Finally, we compare the activation and deactivation dynamics of both the best-performing transcriptional reporter and fluorescent protein to provide a more comprehensive characterization. We observed a 75-fold increase in the activation of the sfGFP fluorescence signal and a 25-fold increase in the activation of the F30-2xdBroccoli signal after 7.5 h. We also observed that the F30-2xdBroccoli RNA aptamer shows slightly faster activation dynamics at the initial stage compared to the relative fluorescence signal of the protein reporter. Regarding the comparison of deactivation dynamics from our experiments, we calculated the decay constant of F30-2xdBroccoli to be two times higher than that of sfGFP. This fact suggests that transcriptional reporters may be a good alternative to protein-based reporters for capturing transient changes in gene expression.

## Results

### Characterizing RNA Aptamers in *E. coli*

To test the utility of RNA light-up aptamers as transcriptional reporters, we implement the Spinach aptamer embedded within a tRNA scaffold,^22,35^ the F30-2xdBroccoli version described by Filonov et al. containing two units of dimeric Broccoli within the F30 scaffold with four binding pockets for the fluorophore,^41^ and the Tornado Broccoli aptamer containing the 49-nt-long Broccoli aptamer flanked by two twister ribozymes.^36^ The ribozymes are intended to undergo catalytic cleavage. Subsequently, an RtcB ligation can circularize the RNA, which should result in RNA aptamers with high stability and expression levels. To characterize the performance of RNA aptamers in cells, we first investigate their behavior at the population level using dynamic growth experiments in a microplate reader. We aim to examine the detectability and the activation range of RNA light-up aptamers, determine if they offer suitable signal-to-noise ratios, and investigate whether the fluorescence signal can be modulated by the promoter selected.

**Figure 1:**
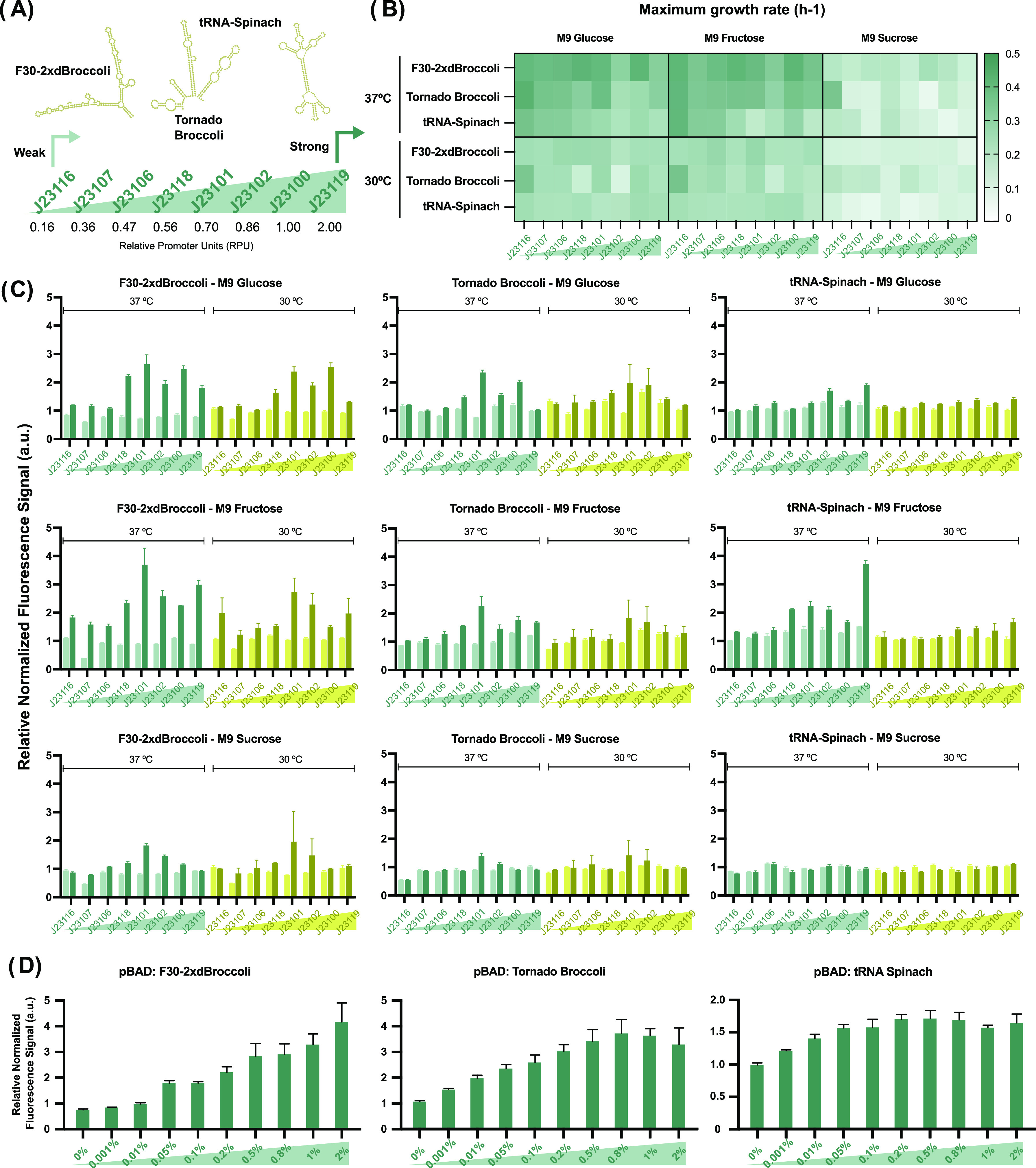
Characterization of RNA light-up aptamers and the promoter library under diverse carbon sources and temperature conditions in a microplate reader. (A) Schematic of the expression system for RNA light-up aptamers with the constitutive promoters used in this study along with their corresponding relative promoter units (RPU). (B) Heat map representing the maximum growth rate values for the RNA aptamer collection and the promoter library in the presence of 160 μM DFHBI-1T in M9 media containing glucose, fructose, and sucrose. Additional data can be found in Figure S3. (C) Performance of F30-2xdBroccoli, Tornado Broccoli, and tRNA-Spinach in distinct media and temperature conditions. Each graph shows the relative fluorescence signal generated by the RNA aptamers across the promoter library under two temperature conditions (37 °C in green and 30 °C in yellow), both in the presence (dark green and dark yellow samples) and absence (light green and light yellow samples) of 160 μM fluorophore. The fluorescence signal is normalized against OD600 and presented as a relative value compared to an internal control (plasmid without expressing the aptamer) in the presence or absence of the fluorophore. Figure S4 shows the results when analyzing fluorescence signals under various conditions during the stationary phase. (D) Evaluation of F30-2xdBroccoli, Tornado Broccoli, and tRNA-Spinach under the arabinose-inducible pBAD promoter. Experiments were conducted at 37 °C using M9 fructose to prevent potential interferences with l-arabinose. Cells were exposed to l-arabinose concentrations ranging from 0 to 2%. The fluorescence signal is normalized against OD_600_ and presented as a relative value compared with an internal control (plasmid without expressing the aptamer) in the presence of the fluorophore and the corresponding inducer concentration. Three biological replicates were utilized, and all error bars represent the standard deviation (s.d.). Statistical analysis was performed, and *p*-values can be found in Tables S1–S4.

The expression levels of the RNA aptamers were tested in DH5α bacterial cells by cloning them downstream of well-characterized constitutive promoters from the Anderson collection in pSEVA-based plasmids.^42−44^ Specifically, the promoters tested, and the strength of the promoters measured in relative promoter units (RPU), are J23116 (RPU = 0.16), J23107 (RPU = 0.36), J23106 (RPU = 0.47), J23118 (RPU = 0.56), J23101 (RPU = 0.70), J23102 (RPU = 0.86), J23100 (RPU = 1), and J23119 (RPU = 2; Figure 1a). We attempted to conduct this characterization in alternative strains of *E. coli*, such as the DH10B strain; however, our efforts were unsuccessful due to the identification of mutations within the promoter regions in this strain.

We first investigated the optimal concentration of fluorophore DFHBI-1T for the activation of the RNA aptamers. DFHBI-1T has previously been used in low concentrations for microscopy experiments, but not in dynamic growth experiments in microplate readers.^45^ We examined the fluorescence signals obtained for the F30-2xdBroccoli RNA aptamer at different concentrations of DFHBI-1T ranging from 0 to 200 μM. This aptamer was chosen, as it has four binding pockets for the fluorophore and therefore sequesters the most fluorophore molecules per transcript. We observed a clear fluorescence signal relative to no-dye and no-aptamer controls at 40 μM DFHBI-1T and above (Figure S1a). The addition of the fluorophore with no RNA expression induces little change. The increase of DFHBI-1T from 40 to 200 μM led to a slight increase in the fluorescence signal per cell, probably due to a larger number of aptamer–dye complexes being formed. However, this increase in the fluorescence signal was not clearly proportional to the concentration of dye used, and we found an optimal concentration of DFHBI-1T between 80 and 160 μM DFHBI-1T. In addition, we examined the effect of the fluorophore in bacterial cells and confirmed that DFHBI-1T does not seem to be cytotoxic, as varying its concentration did not have any effect on cell growth^19^ (Figure S1b).

Next, we evaluated the fluorescence levels produced when the RNA aptamers are expressed using different pSEVA-based copy plasmids using pRO1600_ColE1, p15a, and pBBR1 as origins of replication (Figure S2). Significant activation of the fluorescence signal (*p*-value <0.05) is only detected when the RNA aptamers are expressed in a high-copy plasmid (pRO1600_ColE1). It should be noted that for the F30-2xdBroccoli aptamer, significant differences are observed across all three origins of replications when the RNA aptamer is expressed with a medium- and high-strength promoter. Based on these results, we conducted the following experiments using the pSEVA-based high-copy plasmid.

In order to study the aptamers’ performance in dynamic experiments across diverse conditions, we evaluated the maximum growth rate and the fluorescence signals by testing three distinct carbon sources (M9 media containing glucose, fructose, and sucrose) and two temperatures (37 and 30 °C). This approach was undertaken to explore the variables that could potentially impact their folding and/or expression.

We investigated the impact of expressing RNA light-up aptamers on the growth of *E. coli* under varying environmental conditions of media composition and temperature (Figures 1b and S3). For F30-2xdBroccoli, no significant differences are observed among promoters, but differences are noted among temperatures. At 37 °C, the maximum growth rate on average was 0.4 h^–1^ across the samples, whereas it decreased to 0.26 h^–1^ for cultures grown at 30 °C when glucose and fructose were used as carbon sources. Interestingly, sucrose utilization resulted in a noteworthy reduction in the maximum growth rate, nearly halving the growth rate observed in the previous conditions. For Tornado Broccoli samples, the average of the maximum growth rate for cultures grown in glucose and fructose was 0.35 h^–1^ at 37 °C and around 0.25 h^–1^ at 30 °C. Similar to F30-2xdBroccoli, a notable impact on the maximum growth rate is observed when sucrose is employed as the carbon source, with growth rates of around 0.17 h^–1^ at both temperatures. Finally, it is worth noting that the tRNA-Spinach RNA aptamer at 37 °C exhibits the most substantial impact on cell growth, where increasing promoter strength leads to a significant reduction in the maximum growth rate. This effect is observed in both glucose- and fructose-containing media. In sucrose-containing media, the growth rate of tRNA-Spinach samples is impaired at both temperatures.

To compare the performance of the different aptamers, we carried out time-course experiments and calculated the fluorescence signal at the maximum growth rate, normalizing it by the absorbance at 600 nm. This time point was selected because the maximum growth rate and the maximum number of free RNA polymerases in the cell are positively correlated, which makes it a good indicator of the availability of transcriptional resources for the expression of RNA aptamers.^46^ Samples are normalized by an internal control, the absence of the RNA aptamer, and the presence or absence of fluorophore DFHBI-1T.

The fluorescence signal emitted by the F30-2xdBroccoli RNA aptamer is significantly higher in the presence of the fluorophore than that in its absence at 37 °C. At lower temperatures, this increase is significant only when cells are grown in glucose or fructose (Table S1). Analyzing the activated signals among the promoters, we observe significant differences between the weak promoters (J23116, J23107, and J23106) and the remaining promoters at both temperatures when the samples are cultured in glucose. The fluorescence signals obtained for the J23101 and J23100 promoters, with RPU 0.70 and 1, respectively, are significantly higher than the signals obtained for J23102 and J23119, with RPU 0.86 and 2, respectively (Figure S4 and Table S2). Using fructose as a carbon source, weak promoters also generate lower fluorescence signals compared to medium and strong promoters. Similarly, the J23101 promoter produces the highest signal, followed by the remaining medium and strong promoters. Similar behavior was observed when F30-2xdBroccoli samples were cultured in sucrose-containing media. These results indicate that the fluorescent output can be modulated by the promoter strength. Despite the fact that we could not detect a significant difference between medium and strong promoters, the results are suggestive of clear differences in the fluorescence output between weak and strong promoters for the six conditions tested.

A similar but slightly weaker response is observed for the Tornado Broccoli RNA aptamer (Figure 1c). In the presence of DFHBI-1T, a significant increase in the fluorescence signal is observed for the samples grown in the presence of DFHBI-1T compared to those grown in the absence of the fluorophore at 37 °C. This result differs from what was obtained at 30 °C, where significant activations were only observed for the J23101 promoter for the three carbon sources (Table S1). As with F30-2xdBroccoli, we observed significant differences between weak promoters (J23116, J23107, and J23106) and medium to strong promoters in both glucose and fructose media, and J23101 (RPU 0.70) exhibited the highest fluorescence signal. In sucrose-containing media, significant fluorescence signal activation was observed only using J23101 and J23102 promoters (Table S3).

As can be observed in Figure 1c, the fluorescence signals detected for tRNA-Spinach report lower fluorescence signals under the tested conditions. The fluorescence signal emitted by tRNA-Spinach is significantly higher in the presence of DHBI-1T than in its absence at 37 °C using M9 glucose and fructose. At 30 °C, only M9 Glucose produced a significant fluorescence activation (Table S1). As for the other two RNA aptamers, there are differences between weak and the remaining promoters, and we observed enhanced results using M9 fructose at 37 °C (Table S4). Figure S4 shows the fluorescent activation of the RNA light-up aptamers in the stationary phase.

Considering these results, we observe that decreasing the temperature does not improve the folding of RNA aptamers or enhance the fluorescence signal of the samples. In fact, improved results are obtained when culturing cells at 37 °C. Additionally, we provide evidence supporting the modulation of RNA aptamer expression through the use of different promoters. Figure S5 illustrates the correlation between promoter strength and the fluorescence signal emitted by the aptamer under each of the tested conditions. The results indicate that tRNA-Spinach is the only RNA aptamer displaying a significant positive correlation between promoter strength and the fluorescence signal obtained in both fructose and glucose M9 media, both at 37 and 30 °C. For F30-2xdBroccoli and Tornado Broccoli, the strongest promoter does not consistently produce the highest fluorescence signal. In addition, we observe a positive and significant correlation between the results obtained at both temperatures.

To gain further insights into these promoters, we expressed the RNA aptamers under the arabinose-inducible pBAD promoter (Figure 1d). These results demonstrate an increase in the fluorescence signal as the concentration of l-arabinose increases. A similar trend is observed for Tornado Broccoli, with the maximum signal reached at 0.8% arabinose. Finally, the fluorescence signals obtained for tRNA-Spinach exhibit reduced levels, and signal saturation is observed at an earlier stage. We analyzed the correlation between the fluorescence levels of the protein reporter sfGFP and the RNA aptamers to determine the correlation between both reporters. We observed a statistically significant positive correlation between protein levels and transcriptional reporter activity in both F30-2xdBroccoli and Tornado Broccoli (Figure S6a,b). Notably, among the RNA aptamers examined, F30-2xdBroccoli exhibits the strongest correlation. This correlation analysis was also carried out using constitutive promoters, confirming that F30-2xdBroccoli offers the best correlation between the two variables (Figure S6c,d).

Overall, we have demonstrated that the transcriptional reporters can be detected in microplate readers, and the fluorescent output can be modulated by changing the strength of the promoter that controls their expression. From the promoter library, we observe that there is only a positive correlation between promoter strength and the fluorescence signal for the tRNA-Spinach aptamer. For the other two RNA light-up aptamers, there are no significant differences in the fluorescence signals obtained using medium and strong promoters. However, when the arabinose-inducible promoter is utilized, we observe a correlation between the concentration of arabinose used and the fluorescence signal generated for the three RNA light-up aptamers. When comparing the fluorescence signal emitted by sfGFP and the RNA aptamers under this promoter, we observed a positive and statistically significant correlation. Considering these results, we sought to establish a correlation between relative gene expression and relative fluorescence signals in arabinose-induced samples (Figure S7). RT-qPCR confirmed the successful expression of RNA aptamers following the addition of l-arabinose, validating our intended experimental setup. For the RNA aptamer F30-2xdBroccoli, we observed a substantial 2.5-fold increase in relative expression in the 0.02% induced sample compared to the noninduced sample. This increase in expression increased 13-fold in the 0.2% induced sample and 150-fold in the 1% induced sample. When assessing tRNA-Spinach, we observed a 2.5-fold increase in expression in the highest induced samples, while the lower induction levels did not exhibit a significant increase in gene expression. Unfortunately, our attempts to assess tetra-broccoli were not successful. This RNA aptamer may need further optimization for RT-qPCR as the resultant fragment after ribozyme cleavage falls below the 100bp threshold, potentially complicating the detection process. However, correlation analysis revealed no significant correlation between gene expression and fluorescence signals for both studied RNA aptamers. These results could be explained by the fact that the fluorescence signals produced by the RNA reporters are indicative of the number of properly folded RNA molecules rather than the number of transcripts expressed. The correct folding and structural stability of the RNA structures are likely crucial for fluorescence emission by the RNA aptamers. However, an extensive investigation to correlate the RNA levels and the RNA fluorescent output is beyond the scope of this work. We consider that the overall usefulness of these transcriptional reporters is determined by whether the combination of the RNA light-up aptamer and promoter used produced a clear fluorescence signal that is sensitive enough to be captured by common detection systems such as microplate readers.

### Investigating Variability in Gene Expression at Single-Cell Levels

In the previous section, we investigated the performance of RNA light-up aptamers in microplate readers. However, transcription is a stochastic biological process that leads to high variabilities in the expression of RNA between cells,^47,48^ and bacterial growth can have a significant impact on the transcriptional reporters, as the number of RNA polymerases is growth rate dependent.^49^ In addition, metabolite levels, dynamics, and cell states lead to cell-to-cell differences that can affect the function of RNA-based circuits at the population level. In order to gain more insights into the performance of light-up RNA aptamers, we decided to study RNA dynamics at the single-cell level using flow cytometry. By scrutinizing thousands of cells per second, this technique enables us to provide complementary information to the results obtained for the population-based experiments. We analyze not only the fluorescence signal from the different combinations of RNA aptamers and promoters but also how variable the RNA expression is among the populations. This aspect is particularly relevant, as it allows us to examine whether the fluorescence signal in the sample is homogeneous or, on the contrary, the expression of RNA light-up aptamers produces heterogeneous populations.

**Figure 2:**
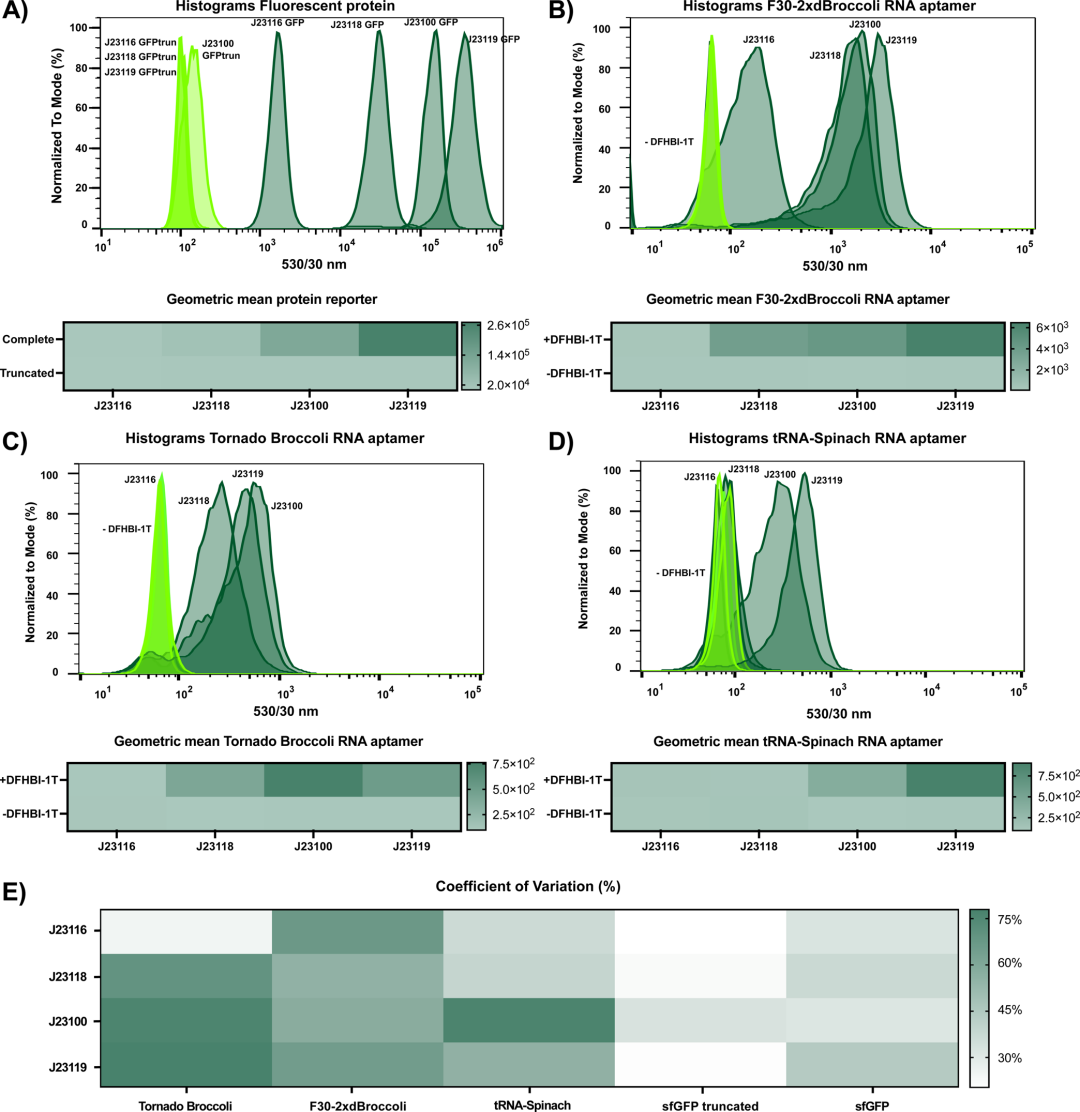
Characterization of RNA light-up aptamers using flow cytometry. (A) Expression analysis of both functional and truncated versions of sfGFP. The histogram shows the population distributions, and the heat map indicates the geometric mean calculated from the histogram of three replicates for each sample. Histograms show modal fluorescence at 530 and 30 nm of a single replicate to facilitate the visualization of the results. (B) Expression analysis equivalent to (A) for F30-2xdBroccoli. The heat map indicates the geometric mean of fluorescence calculated for the samples in the presence and absence of the fluorophore DFHBI-1T. The fluorescence signal is only detected in the presence of the fluorophore, and the signal increases as the strength of the promoter used increases. (C) Expression analysis equivalent to (B) for Tornado Broccoli RNA aptamer. The fluorescence signal is detected when using DFHBI-1T, and the RNA aptamer is expressed under medium and strong promoters. (D) Expression analysis equivalent to (B) for the tRNA-Spinach RNA aptamer. Similar to F30-2xdBroccoli, the fluorescence signal is only detected in the presence of the fluorophore, and the signal increases as the strength of the promoter used increases. However, there is no activation of the fluorescence signal when the aptamer is expressed under weak or medium promoters. (E) Dispersion analysis for proteins and RNA reporters. The heat map displays the results for the coefficient of variation (CV) for sfGFP, truncated sfGFP, and in the presence and absence of DFHBI-1T for the RNA light-up aptamers. Strains expressing both functional and truncated versions of sfGFP show less dispersion than RNA light-up aptamers, and among the latter, the Tornado Broccoli aptamer displays higher dispersion compared to the other two aptamers. Biological replicates can be found in Figure S9. Statistics and ANOVA analysis for the samples can be found in Tables S5–S8.

We assess the performance and heterogeneity of some of the RNA fluorescent reporters and compare them to a fluorescent protein by using flow cytometry. From the previous section, we selected four constitutive promoters J23116 (RPU = 0.16), J23118 (RPU = 0.56), J23100 (RPU = 1), and J23119 (RPU = 2) driving the production of sfGFP and a version of truncated sfGFP that was assembled as controls. The geometric means of the fluorescence signals were determined after 1 h of incubation to provide an overall measure of performance, and the histograms were plotted to study the dispersion of each population. As expected, the samples expressing the truncated version of the fluorescent reporter do not produce any fluorescence signal, whereas the samples expressing sfGFP do, and they correlate with the strength of the promoter (Figures 2a and S8).

Using the same approach, we confirmed the correlation observed in the previous section between fluorescence signals and the promoters used to express the RNA light-up aptamers. This technique is sensitive enough to observe significant differences that were not emphasized in population-based experiments, such as the results for some weaker promoters. For F30-2xdBroccoli, we observe a correlation between fluorescence signals and promoters’ strengths and also a significant difference between medium and strong promoters (*p*-value <0.05) that was not observed in the previous bulk experiments (Figure 2b). For Tornado Broccoli, the strongest promoter J23119 produces a fluorescence signal that is not significantly different from J23118 (*p*-value >0.05), corroborating the results observed in the previous section (Figure 2c). For tRNA-Spinach, the highest fluorescence signal can be observed by using strong promoters (Figure 2d). In this case, no such significant activation is observed for the promoters J23116 and J23118, as also observed in the previous bulk measurement result for glucose-containing media (Figure 1d). Considering the toxicity effects of the tRNA-Spinach aptamer, we hypothesize that the fluorescence signal may be dependent on the cell growth phase at the time of measurement. This disparity between the results obtained in this section and the previous ones can be explained by the cell growth and incubation time of the fluorophore during the experiments. These strains were checked multiple times by sequencing to be confident in the correct sequence. The histograms of three biological replicates in the presence and absence of fluorophore DFHBI-1T can be found in Figure S9.

We carried out a dispersion analysis to compare the heterogeneity or noise in gene expression between RNA light-up constructs and protein reporters. We calculate the coefficient of variation (CV) and observe that cells expressing the fluorescent protein (sfGFP) produce a less dispersed population than cells producing RNA light-up aptamers (Figure 2e). The values obtained for this dispersion analysis can be found in Tables S5–S8. We suggest that the variability of the RNA fluorescent reporters is higher than that of fluorescent proteins, which results in more heterogeneous populations. This variability observed at the single-cell level could be due to stochasticity of the transcriptional process.^50^ This effect causes cell populations to exhibit what is known as phenotypic variability and should be considered for the implementation of these RNA-based reporters. Other factors could be the asymmetric portioning of cellular components during cell division^51^ or the fact that these RNA molecules may also be interacting with other RNA strands that are present at variable concentrations in the cells. Their short lifetime and lower counts make these effects stronger for RNA molecules. However, there are also extrinsic factors that may contribute to the higher RNA variability of RNA light-up aptamers compared to sfGFP controls. These include diffusion of the fluorophore through the membrane and its concentration within the cell. Under the microscope, it can be observed that not all cells are activated, and those that are activated usually produce different fluorescence intensities.^34^

In conclusion to this analysis, RNA light-up aptamers can be detected and correlated to the strength of the promoters not only at the population level but also at the single-cell level. We also confirm that F30-2xdBroccoli is the RNA light-up aptamer showing the best performance among all those tested. Using flow cytometry, we performed a dispersion analysis at the single-cell level that revealed that transcriptional reporters produced more variability than protein-based reporters. This effect could be, at least in part, due to the intrinsic stochasticity of transcriptional reporters.

### Dynamical Behavior of RNA Aptamers

Our previous results discussed above reveal the potential of RNA light-up aptamers as transcriptional reporters for population and single-cell experiments. Although fluorescent proteins are brighter, one possible case for RNA aptamers is to report rapid changes in cellular conditions. We investigated the temporal variation of RNA aptamer fluorescence by using the well-characterized arabinose-inducible pBAD promoter.^52^ Unlike constitutive promoters, an inducible system allows temporal control of expression, which makes it more suitable for studying the dynamics of RNA aptamers.

To explore whether we could find evidence of faster dynamics for transcriptional reporters than protein-based reporters, we grew the strains in rich media and either 0 or 1% l-arabinose depending on whether the cells needed to be inactivated or activated, respectively. Then, both samples were washed and resuspended in fresh media with 0 or 1% l-arabinose. Therefore, four different possibilities were analyzed: 0–0% l-arabinose as a negative control, 1–1% l-arabinose as a positive control, 0–1% l-arabinose to study the activation of F30-2xdBroccoli and sfGFP, and 1–0% l-arabinose to study the loss of signal of the RNA aptamer and sfGFP. Using flow cytometry, we determined the geometric mean of the fluorescence signals at different time points. In the previous section, we observed that aptamers can result in heterogeneous and bimodal populations where there are nonactivated and activated cells. To study the dynamics of RNA light-up aptamers, we decided to calculate the deactivation/activation rate and the change in fluorescence signal by considering only the activated population. Therefore, this enables us to monitor the loss/gain of the fluorescence signal without being affected by the nonfluorescing population that will change over time for the activation and deactivation experiments. Note that including this population would make the observed effects stronger.

**Figure 3:**
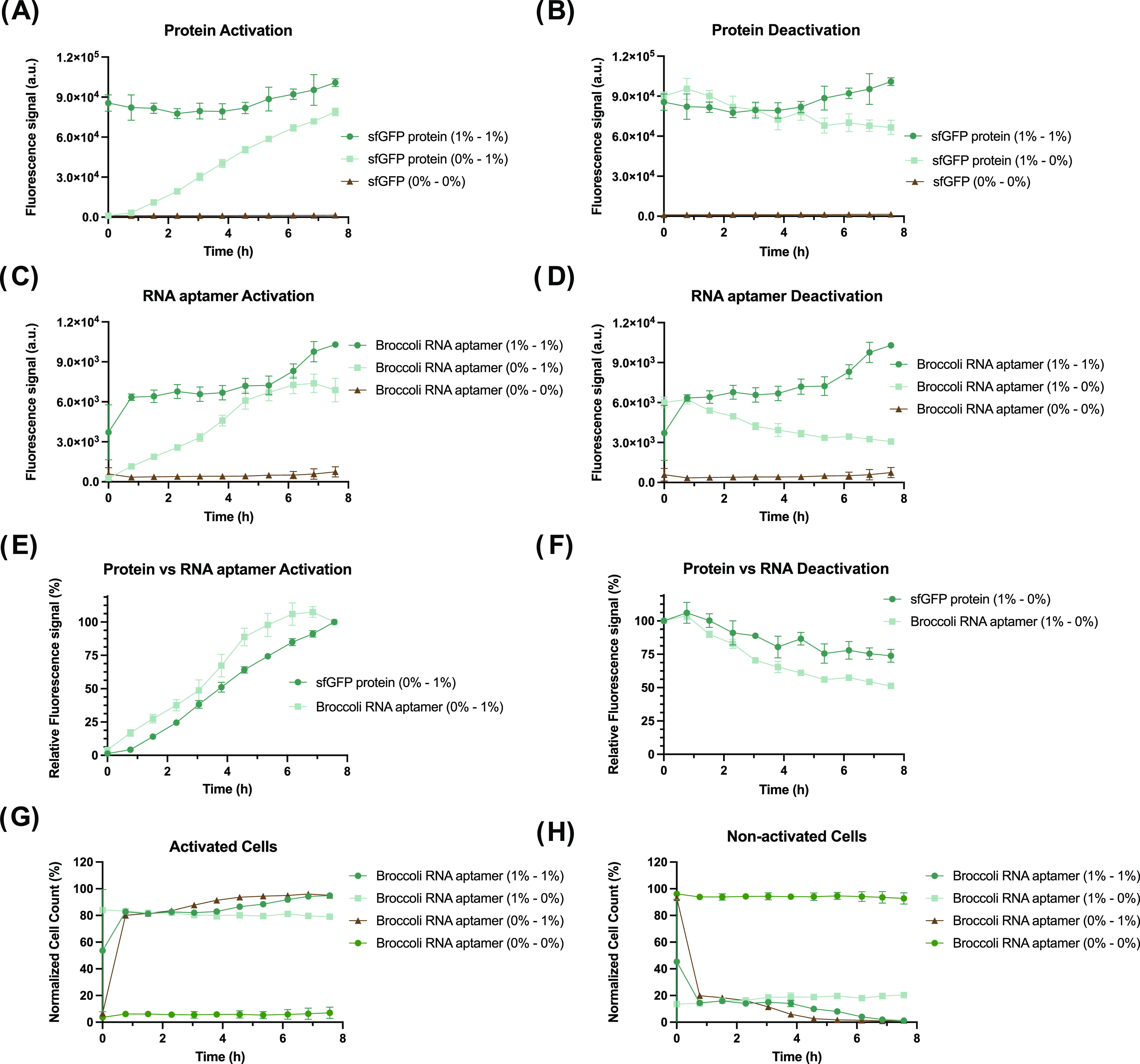
RNA light-up aptamer kinetics. Arabinose-induced expression of sfGFP and F30-2xdBroccoli aptamers. Cells can be exposed to 0 or 1% of l-arabinose, and it is indicated whether the cells are preinduced and/or induced at the initial time point with the % values in brackets. Data show the geometric mean of the fluorescence signal determined by the 10,000 cells in the flow cytometer and across three biological replicates. The positive control represents the reporter being continuously expressed (1–1%) and the negative control is the reporter with no inducer (0–0%). (A) Protein signal activation dynamics. The sample sfGFP (0–1%) in light green shows an increase in the fluorescence signal after exposure to the inducer. Positive control is represented in dark green, and negative control in gray. (B) Protein signal deactivation dynamics. The sample sfGFP (1–0%) in light green shows a decrease in the fluorescence signal over time. Positive control is represented in dark green and negative control in gray. (C) RNA light-up aptamer signal activation dynamics. Same as in (A), the sample F30-2xdBroccoli (0–1%) in light green shows an increase in the fluorescence signal over time. Positive control is represented in dark green and negative control in gray. (D) Decay of the RNA light-up aptamer signal. Same as in (B), the sample F30-2xdBroccoli (1–0%) in light green shows a decrease in the fluorescence signal over time. Positive control is represented in dark green and negative control in gray. (E) Comparison of protein and RNA aptamer activation dynamics. The fluorescence signal from the RNA light-up aptamer immediately starts rising, whereas sfGFP has a delay of approximately 1 h before increasing. Data show the normalization of the fluorescence signal for the initial value. (F) Comparison of protein and RNA aptamer deactivation dynamics. Data show the normalization of the fluorescence signal for the final value. (G) Cell counts of the positive population for F30-2xdBroccoli samples are normalized to give 100% as the final value. (H) Same procedure as that for (G) but for negative populations of F30-2xdBroccoli samples.

We first examine the protein dynamics for sfGFP. Upon activation (0–1%), noninduced cells produce a 75-fold increase in the fluorescence signal after 7.5 h in the presence of 1% l-arabinose, whereas the fluorescence signal for the positive control (1–1%) is initially high and slightly increases over time (Figure 3a). For the deactivation (1–0%), we observe a slow drop in the sfGFP fluorescence signal. However, this reduction in fluorescence is only statistically significant after 5 h from the removal of the inducer (Figure 3b).

We then examine the F30-2xdBroccoli dynamics under the same conditions. In the presence of the inducer, a 25-fold increase in the fluorescence signal was detected for the postinduced sample (0–1%) after 7.5 h. We also observed a significant increase for the preinduced sample (1–1%) during the first hour, which is probably due to the binding/unbinding process of the fluorophore during the wash step (Figure 3c). For the deactivated RNA aptamer sample (1–0%), the fluorescence signal decreased by approximately 50% after 7.5 h in the absence of the inducer (Figure 3d). Experimental data can be found in Table S9, and the histograms for each time point, condition, and biological replicates can be found in Figures S10–S13.

Comparing the dynamics of protein and aptamer activation, we can observe that the fluorescence signal from the RNA light-up aptamer immediately starts rising, whereas sfGFP has a delay of approximately 1 h before increasing (Figure 3e). It is important to note that the F30-2xdBroccoli aptamer had faster signal activation than sfGFP across all biological replicates. Similarly, by comparing the deactivation dynamics between proteins and aptamers, we can observe that the signal of the fluorescent aptamer decays faster than the signal of the fluorescent protein (Figure 3f). The half-life, which is defined as the amount of time *t* a given quantity *N*_0_ takes to decrease to half of its initial value, can be calculated according to the formula *t*_1/2_*=* −*t*·ln(2)/ln(*N*/*N*_0_). By applying this formula, we roughly estimate that the half-life of sfGFP is >17 h with a decay constant of λ = 1.1 × 10^–5^ s^–1^ and the half-life of F30-2xdBroccoli is <8 h with a decay constant of λ = 2.5 × 10^–5^ s^–1^.

Finally, we analyzed the number of cells that were present both in the active and inactive populations for F30-2xdBroccoli (Figure 3g,h). For the deactivation study where the preinduced samples were washed (1–0%), the number of activated cells decreased from 84 to 79% and the number of inactivated cells increased from 13.5 to 19.2%. For the activation experiments where the samples were postinduced (0–1%), the number of activated cells increased from 7 to 95%, whereas the number of inactivated samples decreased from 93 to 1% (Table S10).

In this section, we provide initial evidence that the activation and deactivation dynamics for F30-2xdBroccoli might be faster than those for sfGFP. We observed a 75-fold increase in the activation of the sfGFP fluorescence signal and a 25-fold increase for the F30-2xdBroccoli signal after 8 h. We found that the F30-2xdBroccoli RNA aptamer shows slightly faster activation dynamics compared to the relative fluorescence signal of the protein reporter over 7.5 h. Regarding the comparison of deactivation dynamics from our experiments, we calculated the decay constant of F30-2xdBroccoli to be twice as fast as that of sfGFP.

## Discussion and Conclusions

Protein-based fluorescent reporters are widely used in molecular and cellular biology. However, recent advances in the RNA nanotechnology field have enabled the emergence of alternative reporters based on RNA molecules. These transcriptional reporters are potentially a more convenient option than protein-based reporters for certain applications, such as reporting on RNA-based circuits or circuits with fast dynamics.

In this work, we explore and characterize RNA light-up aptamers as nonprotein-based reporters in *E. coli*. We prove that DFHBI-responsive RNA light-up aptamers can be implemented as fluorescent transcriptional reporters, and we observe a correlation between promoter strength and the fluorescence signal. To our knowledge, this is the first time an application of the Tornado system has been characterized in more detail in *E. coli*, and our work describes for the first time the dynamics of F30-2xdBroccoli, Tornado Broccoli, and tRNA-Spinach in time-course experiments using microplate readers. We observe that the fluorescence signal produced by the RNA light-up aptamers is approximately 10× lower than for sfGFP, but the signal is clear enough to be detected in microplate readers. We also performed a dispersion analysis of the RNA-based reporters compared to protein-based reporters to study the suitability of these structures as fluorescent reporters. We concluded that the output is more variable for transcriptional reporters than for protein-based reporters. Finally, we demonstrate experimentally that the signal activation and deactivation dynamics seem faster for RNA light-up aptamers than those for protein reporters, consistent with expectations.

We observed that the F30-2xdBroccoli RNA aptamer produced the highest fluorescence signal and signal-to-noise ratio. The stability of the structure described in the literature^23^ and the existence of four binding pockets for the fluorophore could explain this performance. For the Tornado Broccoli RNA aptamer, the results from both population and single-cell experiments indicate that even with the strongest promoter, the fluorescence signals are not very bright. It should be considered that Tornado Broccoli binds to one molecule of dye per transcript, whereas F30-2xdBroccoli binds to four units per transcript. Further research is needed on the circularization of larger molecules and more complex secondary structures in order to use the circularization strategy with the F30-2xdBroccoli aptamer version. Furthermore, additional testing can be done with alternative methods to circularize RNA structures^37,38^ that could avoid a potential misfolding of the RNA secondary structures. We can conclude that Tornado Broccoli is not a very effective reporter in these conditions due to its low fluorescence signal but has the potential to be improved to take advantage of its previously reported stability and its ability to be modified to express more complex secondary structures. We also observed that the expression of the tRNA-Spinach aptamer affects cell viability. This effect can determine the performance of this aptamer in microplate readers and single-cell experiments. Regarding this aptamer, the disparity observed between the fluorescence results obtained in the bulk experiment and the flow cytometer can be explained by the incubation times of the fluorophore as well as the growth times and, consequently, the production of the RNA aptamer. Further tests could be conducted to analyze this effect and understand how the activation of the tRNA-Spinach aptamer is affected by these factors.

We provide evidence that F30-2xdBroccoli dynamics are faster than those of sfGFP reporters. This fact suggests a role for RNA reporters, despite the other advantages of GFP, in reporting on rapidly changing signals.

This first characterization allows us to understand the dynamics of an RNA reporter in comparison to that of the commonly used protein reporter and opens the way to implement more RNA-based circuits in living cells. In this work, we have laid the basis for working with RNA light-up aptamers in both population and single-cell level experiments with potential benefits for several applications. Considering that a cell’s protein and mRNA copy numbers are not always correlated,^49^ this characterization offers the possibility to use RNA aptamers as a suitable alternative to investigate gene expression levels without the need for the translation process. Besides response rates, the other advantage of using RNA reporters is the direct probe of transcription in living systems, which can be beneficial for several applications. For example, RNA-based reporters would be ideal for monitoring the implementation of RNA nanotechnology in living cells and siRNA or the improvement of the CRISPR system.^38,53−55^

## Methods

F30-2xdBroccoli and Tornado Broccoli aptamers were synthesized by Thermo Fisher Scientific and Integrated DNA Technologies (IDT), respectively. The Spinach aptamer inside the tRNA scaffold was obtained from the Ellis lab.^34^ The regulator–promoter sequence AraC-pBAD was obtained from Ceroni et al.^56^ The promoter library and the terminator were assembled scarlessly with the RNA aptamers using Gibson Assembly reactions in the pSEVA141 (pRO1600/Amp) vector.^42^ Constructs were transformed in the *E. coli* DH5α strain (Thermo Fisher Scientific) and verified by sequencing.

For experiments, cells were grown overnight in shaking liquid culture (LB + antibiotic) at 37 °C and 250 rpm in a MaxQ 6000 shaking incubator (Thermo Scientific). Cells were diluted in rich M9 media consisting of M9 salts supplemented with 0.4% casamino acids, 0.25 mg/mL thiamine hydrochloride, 2 mM MgSO_4_, 0.1 mM CaCl_2_, 0.4% carbon source, and the appropriate antibiotic.^56^ Subcultures were incubated at 37 °C until they reached the exponential phase at OD_600_ = 0.2–0.4. For plate reader experiments, cells were diluted to a final OD_600_ = 0.05 in a final volume of 200 μL and transferred to a 96-well flat-bottom plate (Corning Costar, Thermo Fisher Scientific), and the fluorophore DHFBI-1T (Lucerna) was added at the corresponding concentration. Time-course experiments were carried out in a Tecan Spark Microplate Reader (Tecan, Maennedorf, Switzerland) at 37 °C using double orbital shaking at 180 rpm and gain 50. Absorbance was measured at 600 nm, and the fluorescence was detected with the following settings: excitation at 485/20 nm and emission at 535/25 nm. The growth rates per hour were calculated according to the procedure described by Ceroni et al.,^56^ where growth rate at *t_n_* = (ln(OD(*t*_*n*+1_)) – ln(OD(*t*_*n*–1_)))/(*t*_n+1_ – *t*_n–1_). The time point (*n*) at which the cells were at maximum growth was used to analyze the fluorescence signal and normalized to the OD_600_.

Flow cytometry data were obtained using an Attune NxT Flow Cytometer (Thermo Scientific) with the following settings: FSC 10 V, SSC 350 V, and BL1 400 V. For flow cytometry experiments, cells were diluted to OD_600_ = 0.2 to a final volume of 200 μL and were incubated for 1 h at room temperature with 300 rpm shaking (Heidolph-Titramax 101) together with 80 μM fluorophore DHFBI-1T. Cells were washed twice with 1× phosphate-buffered saline (PBS) and resuspended in 200 μL in a 96-well round-bottom microplate (Corning Costar, Thermo Fisher Scientific) prior to the analysis with a flow cytometer. For the reporting dynamics experiments, cells were incubated overnight in shaking liquid culture with rich M9 media supplemented with 0.4% fructose to avoid the strong catabolite repression of AraBAD, 1% l-arabinose (Sigma-Aldrich) if needed, and 80 μM DHFBI-1T if needed. 1 mL of each overnight culture was washed twice with 1× PBS, and cells were resuspended in fresh media with 1% l-arabinose and 80 μM DFHBI-1T if needed. Samples were taken every hour (2 μL of culture diluted in 150 μL of PBS).

Fluorescence data were collected from more than 10,000 cells for each sample, and statistics such as geometric mean, mode, standard deviation, and coefficient of variation were obtained using FlowJo software. Cells were manually gated using FSC-H versus SSC-H to identify cells of interest and FSC-A versus FSC-H to identify singlets. For the dispersion analysis, an additional gate with BL1-H versus FSC-H and the auto gate tool from FlowJo were added to remove outliers that could affect the statistical analysis. One-way ANOVA followed by Tukey’s multiple comparison tests were performed using GraphPad Prism version 9.4.1 for Mac OS X, GraphPad Software, San Diego, California.

For RT-qPCR experiments, cells were grown in rich media at 37 °C and 250 rpm overnight. RNA was isolated from a bacterial culture grown to an OD600 of 1 ± 0.2 using a Thermo Scientific GeneJET RNA (Thermo Fisher Scientific). RNA was quantified by a nanodrop spectrophotometer (Thermo Fisher), and cDNA was generated from each RNA prep using a High-Capacity cDNA Reverse Transcription Kit (Applied Biosystems). qPCR results were normalized to the housekeeping gene 16S. All qPCR primers were designed manually using Benchling (Supporting Table 12). All quantitative PCR (qPCR) reactions were performed in a StepO- nePlusTM Real-Time PCR System (Applied Biosystems) using SYBR Green JumpStart Taq ReadyMix (Sigma-Aldrich).

## Supporting Information Available

Additional experiments for bulk experiments and correlation analyses (Figures S1–S7); experiments involving heterogeneity analysis (Figures S8 and S9); histograms related to dynamics experiments (S10–S13); statistical analyses (Tables S1–S10); and sequences and primers used (Tables S11 and S12) (PDF)
