# Supplementary material for: Evaluating DFHBI-Responsive RNA Light-Up Aptamers as Fluorescent Reporters for Gene Expression
Source: ACS Synth Biol. 2023 Nov 22;12(12):3754–65. doi: 10.1021/acssynbio.3c00599 (PMC10729303; doi:10.1021/acssynbio.3c00599)
Supplement: Supplementary file 1 — sb3c00599_si_001.pdf [file sb3c00599_si_001.pdf]

Evaluating RNA aptamers as fluorescent reporters of gene expression

Alicia Climent-Catala*[1,2,5], Ivan Casas-Rodrigo[4], Suhasini Iyer[1,3], Rodrigo Ledesma-Amaro[1,5] and Thomas E. Ouldridge[1,5]

1 Imperial College Centre for Synthetic Biology, London, SW7 2AZ, U.K.
2 Department of Chemistry, Imperial College London, London, SW7 2AZ, U.K.
3 Department of Life Sciences, Imperial College London, London, SW7 2AZ, U.K.
4 Department of Biosystems Science and Engineering, ETH Zurich, CH-4058 Basel, Switzerland.
5 Department of Bioengineering, Imperial College London, London, SW7 2AZ, U.K.

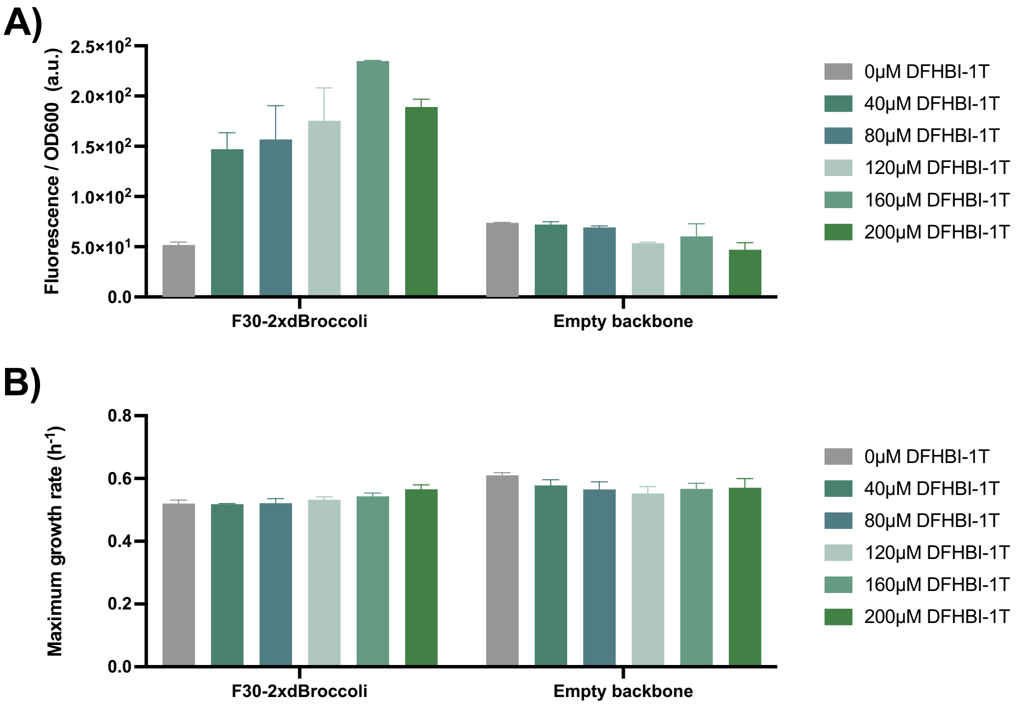

Supplementary Figure 1. **Dose-response of DFHBI-1T in time-course experiments**. F30-2xdBroccoli was used under the strongest promoter J23119 and compared with the signal given by an empty backbone. F30-2xdBroccoli RNA aptamer contains four binding sites allowing this aptamer to sequester more molecules of dye per RNA molecule. a) The data show the fluorescence signal normalised by the OD600 at the maximum growth rate of three biological replicates and b) the maximum growth rate of three biological replicates. All error bars show the s.d.

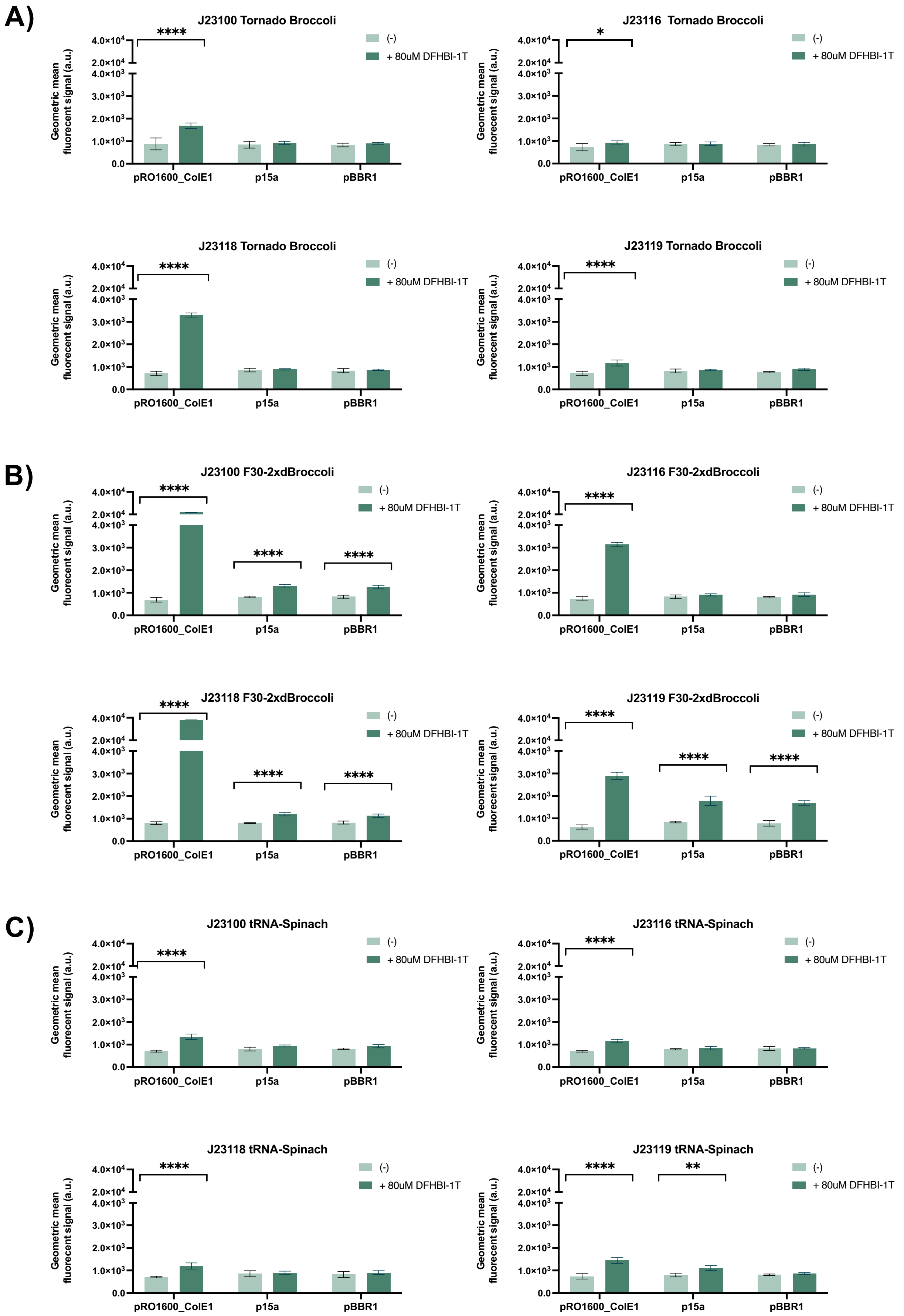
A)
J23100 Tornado Broccoli
J23116 Tornado Broccoli
J23118 Tornado Broccoli
J23119 Tornado Broccoli
B)
J23100 F30-2xdBroccoli
J23116 F30-2xdBroccoli
J23118 F30-2xdBroccoli
J23119 F30-2xdBroccoli
C)
J23100 tRNA-Spinach
J23116 tRNA-Spinach
J23118 tRNA-Spinach
J23119 tRNA-Spinach
Geometric mean fluorecent signal (a.u.)
4.0×10⁴
2.0×10⁴
4.0×10³
3.0×10³
2.0×10³
1.0×10³
0.0
pRO1600_ColE1
p15a
pBBR1
(-)
+ 80uM DFHBI-1T
****
*
**

Supplementary Figure 2. **Comparative analysis of fluorescence levels produced by three RNA aptamers expressed in various pSEVA-Based Copy Plasmids**. RNA light-up aptamers were expressed using weak (J23116), medium strength (J23118), and strong (J23100 and J23119) promoters. Each combination was expressed in different pSEVA plasmids with different origins of replication, namely pSEVA141 (pRO1600_ColE1/Amp), pSEVA261 (p15A/Kam), and pSEVA331 (pBBR1/Cam). **A)** Results for Tornado Broccoli RNA-light up aptamer. **B)** Results for F30-2xdBroccoli RNA light-up aptamer. **C)** Results for tRNA-Spinach RNA light-up aptamer. Cell cultures were grown overnight at 37°C in rich media, followed by the preparation of subcultures in fresh media. These subcultures were incubated at 37°C until the cells reached an OD600 of 0.5-1.2. Subsequently, the cells were incubated with 80 µM of the fluorophore DFHBI-1T for 1 hour, and the resulting fluorescence signals were analysed using the flow cytometer. Two-way ANOVA tests were performed to analyse significant differences in activation between the presence and absence of DFHBI-1T, with a significance threshold set at the 5% level.

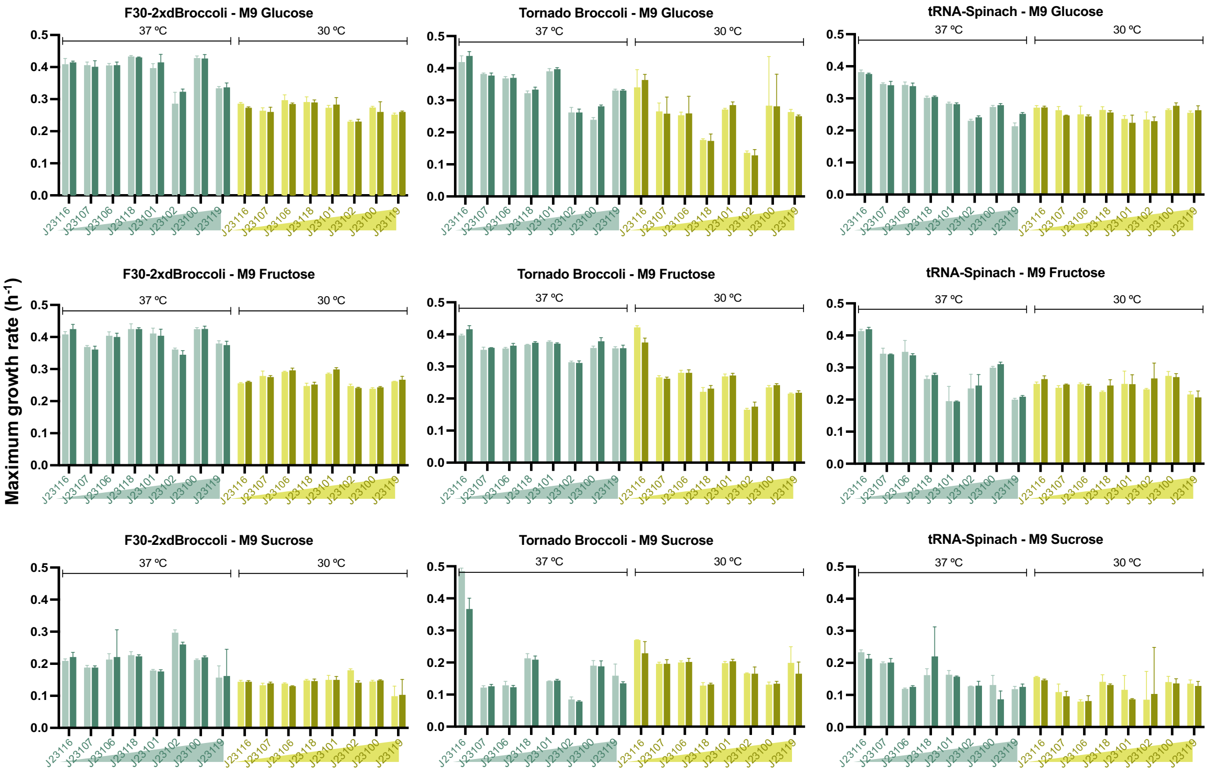

Supplementary Figure 3. Maximum growth rate values for the RNA Aptamer collection and promoter library under varied carbon sources and temperature conditions. Maximum growth rate values obtained for the RNA aptamer collection, including F30-2xdBroccoli, Tornado Broccoli, and tRNA-Spinach, when expressed under a range of constitutive promoters of varying strength. The experiments were conducted under diverse environmental conditions, including growth media with different carbon sources (M9 Glucose, Fructose, and Sucrose) and at two distinct temperatures, 30°C and 37°C. The samples in dark green and yellow indicate that the cultures grew in the presence of 160 µM of the fluorophore DFHBI-1T, while the light-coloured ones indicate growth with 0 µM of DFHBI-1T. Data shown was obtained by performing time course experiments in microplate readers across 3 biological replicates.

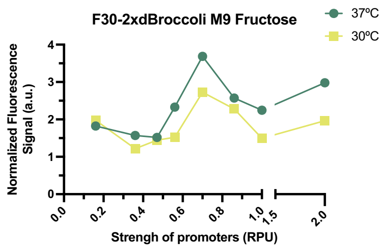

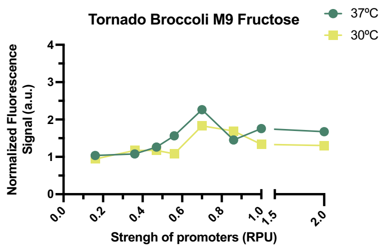

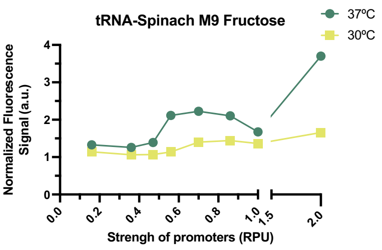

Samples	r^2 coefficient	P value
Prom. strength vs F30-2xdBroccoli 37ºC	0.2869	0.1713
Prom. strength vs F30-2xdBroccoli 30ºC	0.04177	0.6273
F30-2xdBroccoli 37ºC vs 30ºC	0.6782	0.0120

Samples	r^2 coefficient	P value
Prom. strength vs Tornado Broccoli 37ºC	0.2082	0.2557
Prom. strength vs Tornado Broccoli 30ºC	0.09306	0.4625
Tornado Broccoli 37ºC vs 30ºC	0.5483	0.0356

Samples	r^2 coefficient	P value
Prom. strength vs tRNA-Spinach 37ºC	0.8304	0.0016
Prom. strength vs tRNA-Spinach 30ºC	0.7895	0.0032
tRNA-Spinach 37ºC vs 30ºC	0.7376	0.0063

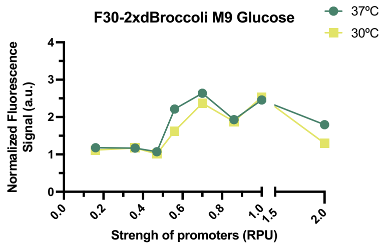

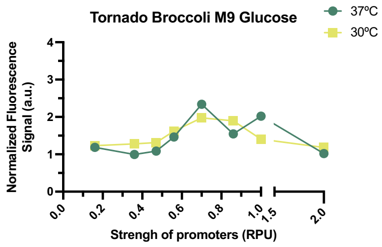

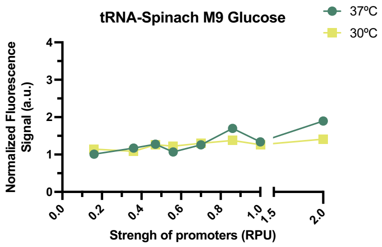

Samples	r^2 coefficient	P value
Prom. strength vs F30-2xdBroccoli 37ºC	0.1269	0.3864
Prom. strength vs F30-2xdBroccoli 30ºC	0.03866	0.6407
F30-2xdBroccoli 37ºC vs 30ºC	0.8421	0.0013

Samples	r^2 coefficient	P value
Prom. strength vs Tornado Broccoli 37ºC	3.785e-007	0.9988
Prom. strength vs Tornado Broccoli 30ºC	0.01078	0.8067
Tornado Broccoli 37ºC vs 30ºC	0.5426	0.0371

Samples	r^2 coefficient	P value
Prom. strength vs tRNA-Spinach 37ºC	0.7682	0.0043
Prom. strength vs tRNA-Spinach 30ºC	0.6275	0.0191
tRNA-Spinach 37ºC vs 30ºC	0.7376	0.0067

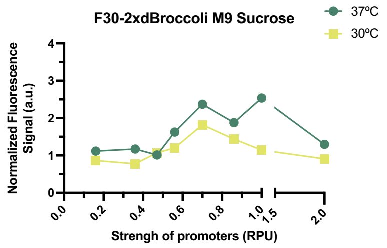

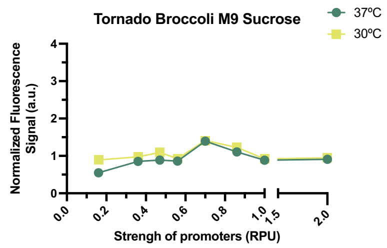

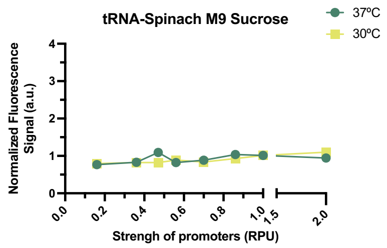

Samples	r^2 coefficient	P value
Prom. strength vs F30-2xdBroccoli 37ºC	0.03866	0.6407
Prom. strength vs F30-2xdBroccoli 30ºC	0.0002247	0.9719
F30-2xdBroccoli 37ºC vs 30ºC	0.5193	0.0437

Samples	r^2 coefficient	P value
Prom. strength vs Tornado Broccoli 37ºC	0.06159	0.5534
Prom. strength vs Tornado Broccoli 30ºC	0.001035	0.9397
Tornado Broccoli 37ºC vs 30ºC	0.8121	0.0022

Samples	r^2 coefficient	P value
Prom. strength vs tRNA-Spinach 37ºC	0.1258	0.3886
Prom. strength vs tRNA-Spinach 30ºC	0.8757	0.0006
tRNA-Spinach 37ºC vs 30ºC	0.1316	0.3771

Supplementary Figure 4. Correlation between Fluorescence Signal and Promoter Strength in RNA Aptamers Across Diverse Media and Temperature Conditions. Correlation analysis between fluorescence signals and promoter strengths for three RNA aptamers (F30-2xd Broccoli, Tornado Broccoli, and tRNA-Spinach) across three different media conditions (M9 Glucose, M9 Fructose, and M9 Sucrose) in both temperature settings (37°C and 30°C). The fluorescence signal obtained from each sample is normalized against the corresponding OD600 value at the maximum growth rate and presented as a relative value compared to an internal control (plasmid without expressing the aptamer) in the presence of the 160 µM DFHBI-1T. Three biological replicates were utilized, and all error bars represent the standard deviation (s.d.). Each graph contains the correlation analysis performed using GraphPrism analysis tool where the correlation coefficient, r, is calculated from the Pearson correlation coefficient and the P value indicates the significance of the correlation.

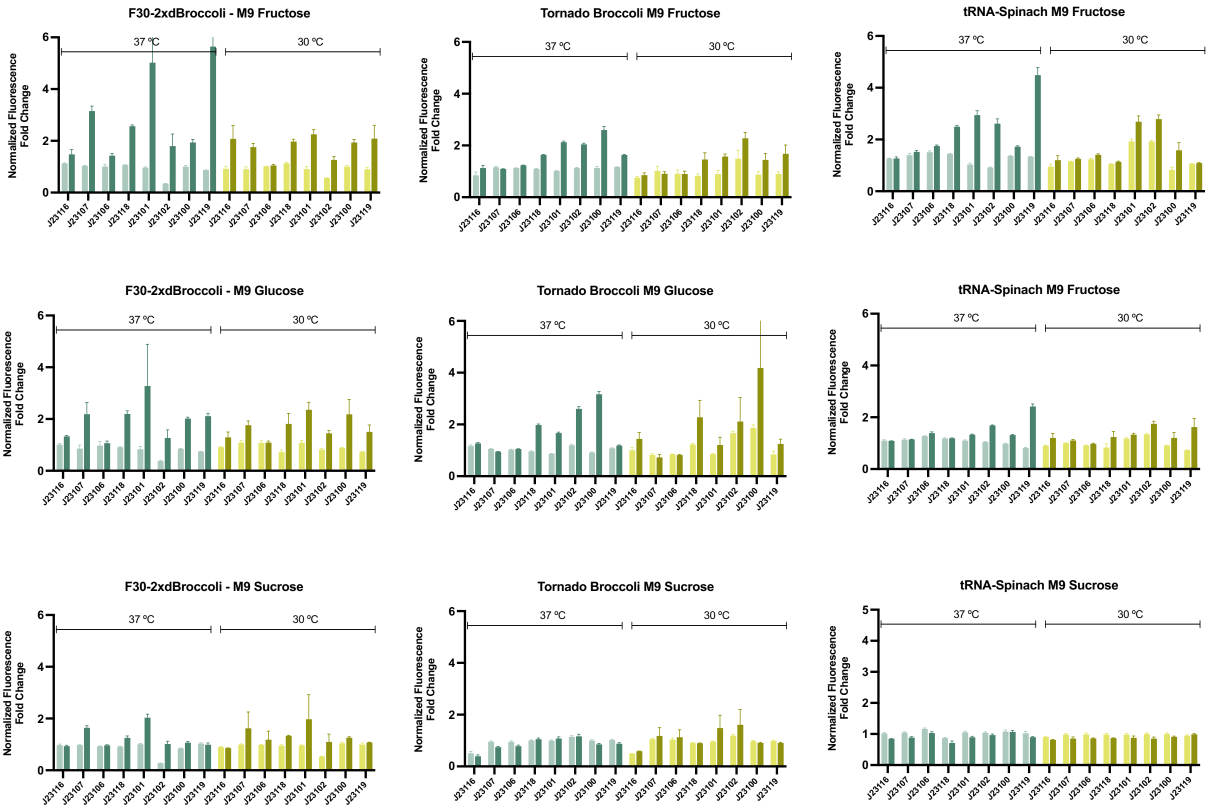

Supplementary Figure 5. **Characterization of RNA light-up aptamers in microplate reader in the stationary phase (after 10h).** Performance of F30-2xd Broccoli, Tornado Broccoli, and tRNA-Spinach in distinct media and temperature conditions. Each graph shows the relative fluorescence signal generated by the RNA aptamers across the promoter library under two temperature conditions (37°C in green and 30°C in yellow), both in the presence (dark green and dark yellow samples) and absence (light green and light-yellow samples) of 160 µM of fluorophore. The fluorescence signal is normalized against OD600 at the maximum growth rate and presented as a relative value compared to an internal control (plasmid without expressing the aptamer) in the presence or absence of the fluorophore. The data throughout the figure show the fluorescence produced at 10h normalised by OD600 of three biological replicates, and all error bars show the s.d.

A) C)

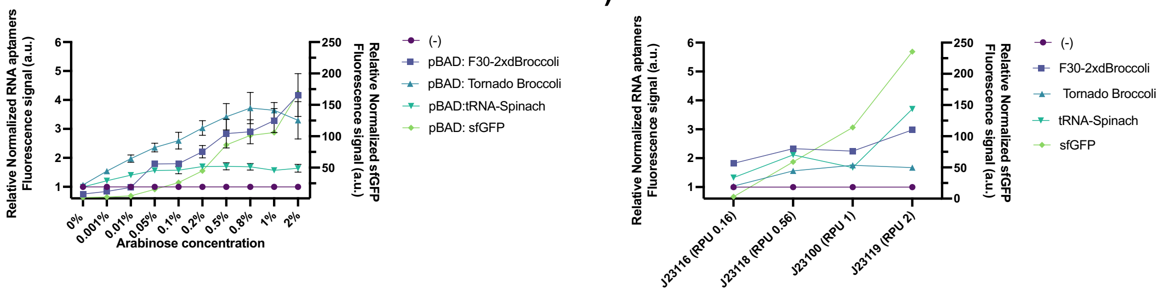

B)

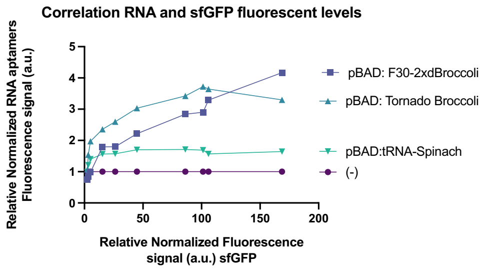

Samples	r^2 coefficient	P value
pBAD:F30-2xdBroccoli vs sfGFP	0.9453	2.501e-006
pBAD:Tornado Broccoli vs sfGFP	0.6605	0.004267
pBAD:tRNA-Spinach vs sfGFP	0.3816	0.05701

D)

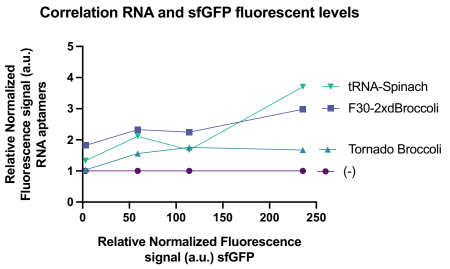

Samples	r^2 coefficient	P value
F30-2xdBroccoli vs sfGFP	0.9099	0.04610
Tornado Broccoli vs sfGFP	0.5173	0.2808
tRNA-Spinach vs sfGFP	0.8258	0.09125

Supplementary Figure 6. Correlation analysis results. A) Relative normalized fluorescence signals for various concentrations of L-arabinose are displayed. The fluorescence signal is normalized against OD600 at the maximum growth rate and presented as a relative value compared to an internal control (plasmid without expressing the aptamer) in the presence of the fluorophore and the corresponding inducer concentration. **B)** Scatterplot illustrating the relationship between the fluorescence levels of the protein reporter sfGFP and the RNA aptamers. The table shows the values for the r2 coefficient obtained from the Pearson correlation coefficient (r) and the p-value, indicating the significance of the correlation between the two studied variables. **C)** Relative normalized fluorescence signals for each reporter under the control of a range of constitutive promoters are presented. **D)** Scatterplot illustrating the relationship between the fluorescence levels of the protein reporter sfGFP and the RNA aptamers. Notably, only the F30-2xdBroccoli RNA aptamer shows a statistically significant positive correlation with the GFP fluorescence signal, with a Pearson correlation coefficient (r) of 0.9539 and a p-value = 0.0461. The sample labelled (-) represents the negative control (plasmid without expressing the aptamer). Mean and standard deviation calculations were based on results obtained from three biological replicates.

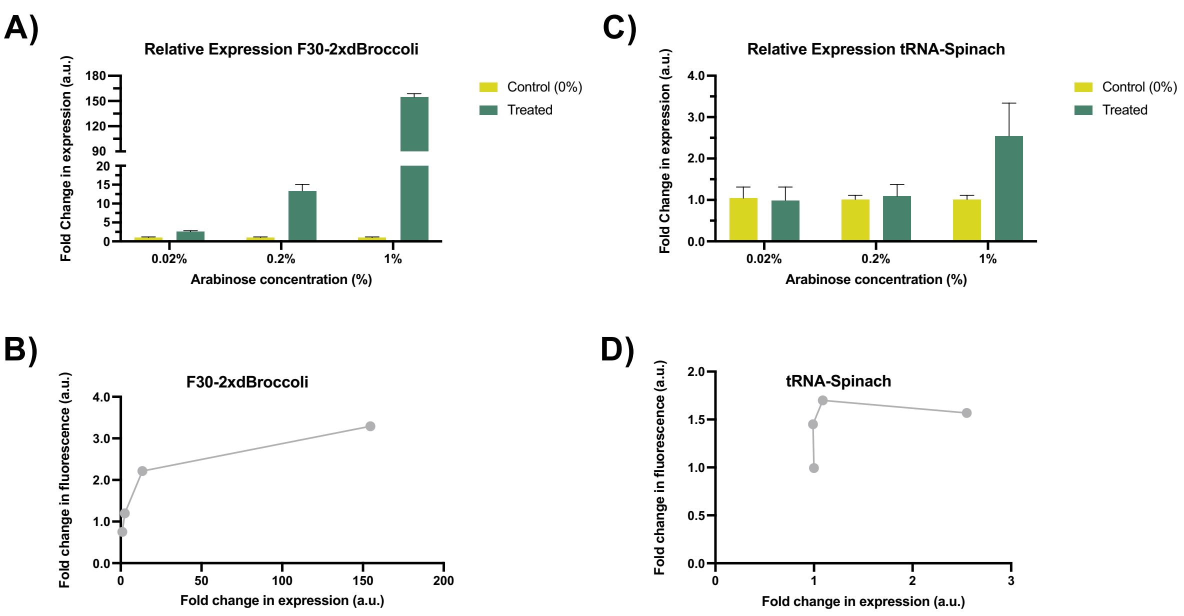

Supplementary Figure 7. Relative expression of the RNA aptamers under the arabinose-inducible promoter. A) Relative expression of F30-2xdBroccoli between non-induced samples (0% L-arabinose) and induced samples (0.02%. 0.2% and 1% L-arabinose). **B)** Scatterplot illustrating the relationship between the F30-2xdBroccoli fluorescence levels vs the protein reporter sfGFP. The correlation between expression and fluorescence shows a Pearson correlation coefficient (r) of 0.8778 and a p-value of 0.1222. **C)** Relative expression of F30-2xdBroccoli between non-induced samples (0% L-arabinose) and induced samples (0.02%. 0.2% and 1% L-arabinose). **D)** Scatterplot illustrating the relationship between the fluorescence levels of the protein reporter sfGFP and the tRNA-Spinach RNA aptamer. The correlation between expression and fluorescence shows a Pearson correlation coefficient (r) of 0.3450 and a p-value of 0.6550. Experimental measurements are relative gene expression levels compared to samples induced with 0% arabinose as determined by RT-qPCR and shown as the mean ± SE from three biological replicates.

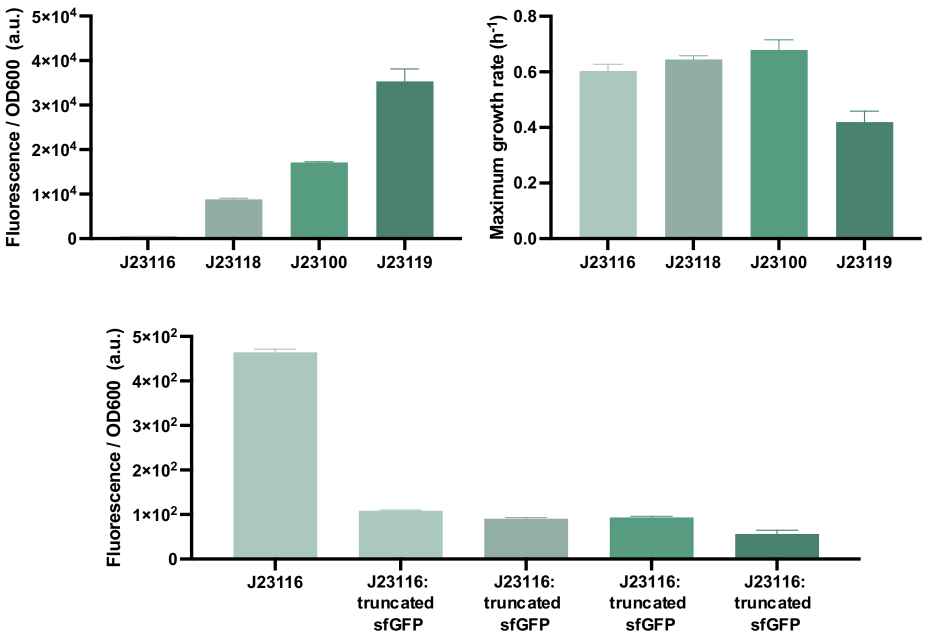

Supplementary Figure 8. Performance of the constitutive promoters with sfGFP. J23116 (RPU=0.16), J23118 (RPU=0.56), J23100 (RPU=1), J23119 (RPU=2) promoters were assembled with the RBS BBa_0034 to drive the constitutive expression of sfGFP in a high-copy plasmid. These promoters were also used to drive the expression of a truncated version of sfGFP with no fluorescent signal to check the activity of the weakest promoter J23116. The figure shows the fluorescent signal normalised by OD600 at the maximum growth rate for three biological replicates, and all error bars show the s.d and the maximum growth rate (h-1) for each sample.

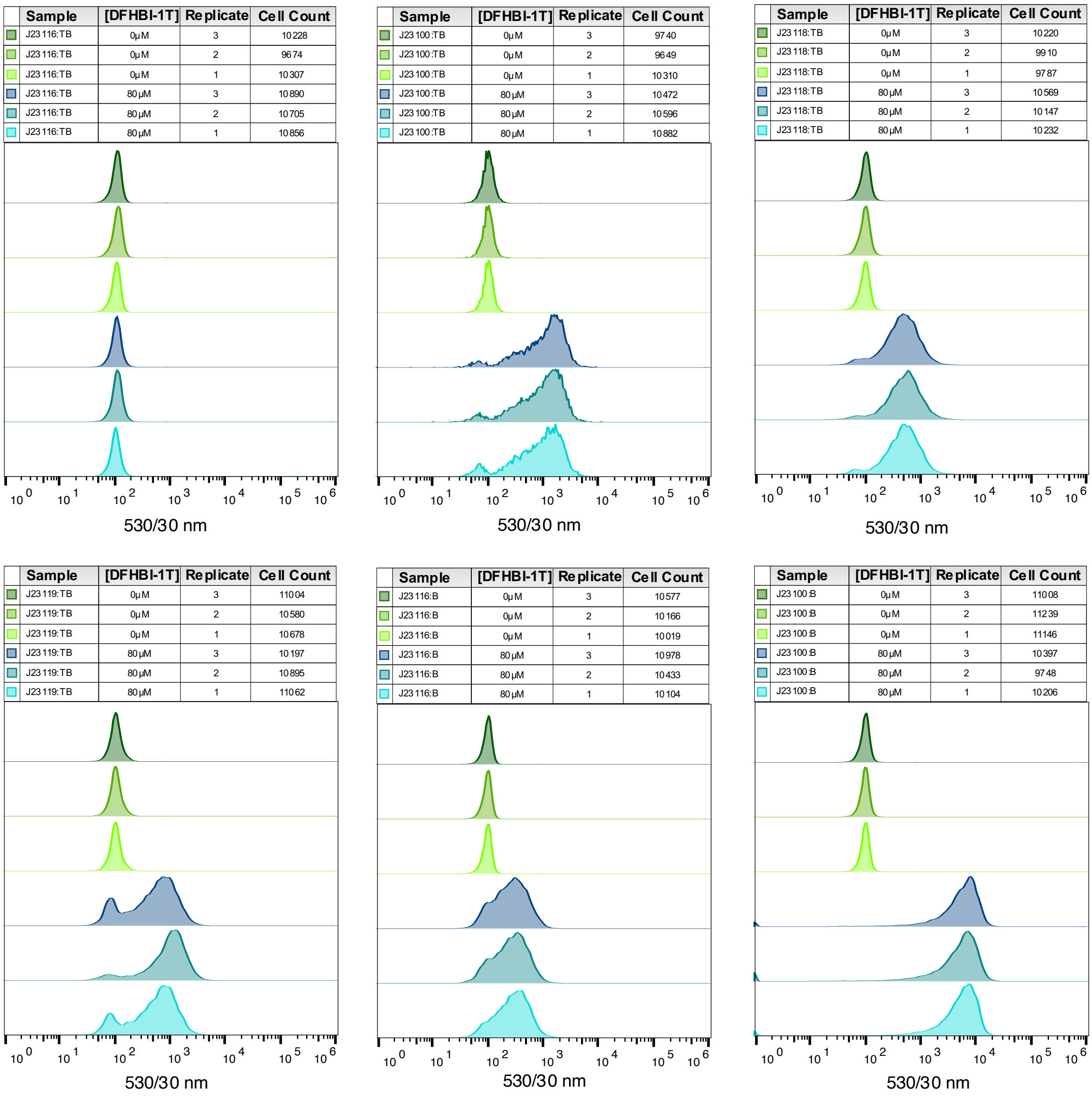

Supplementary Figure 9. Caption in the next page.

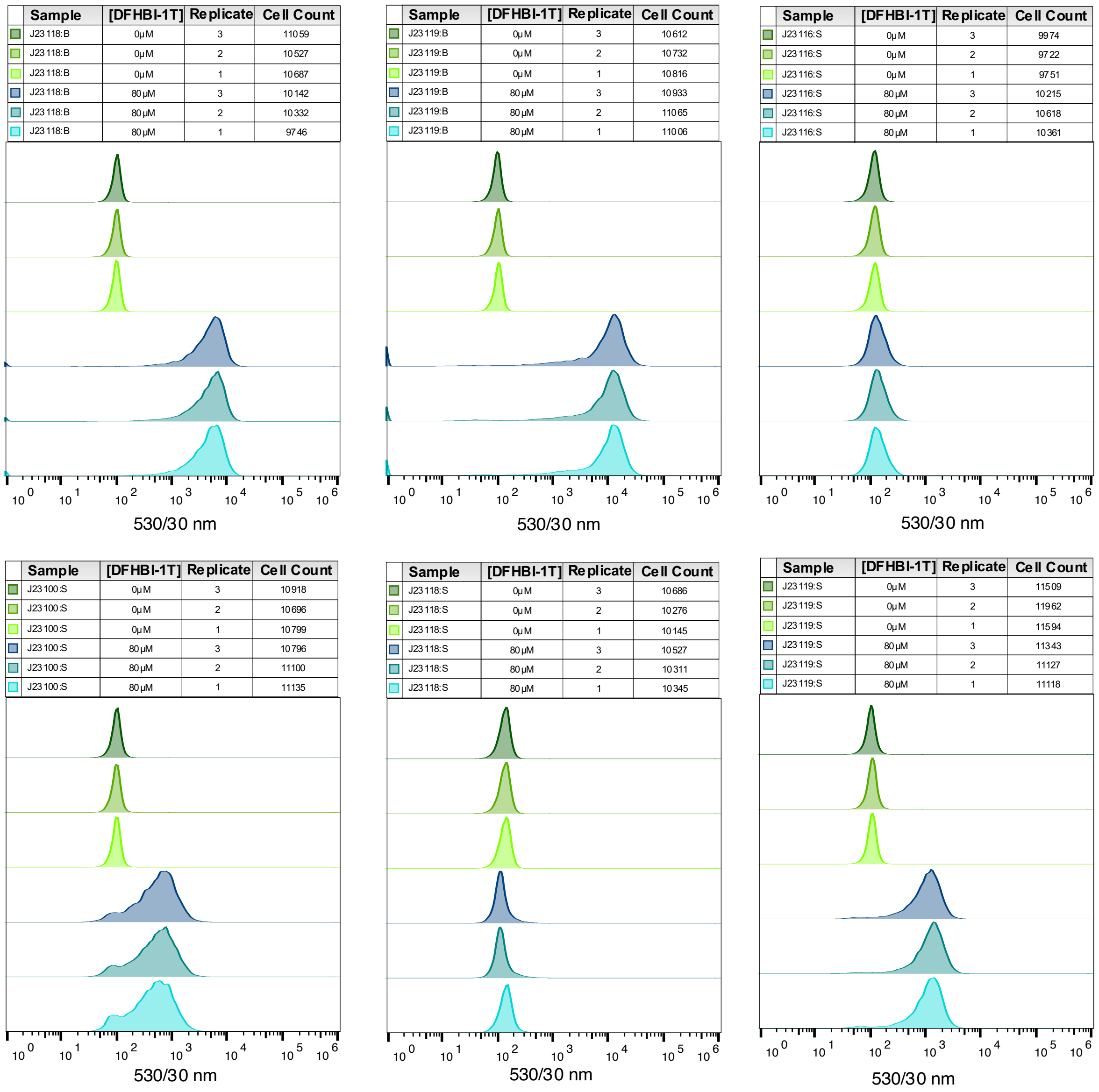

Supplementary Figure 9. Heterogeneity analysis for RNA light-up aptamers. Histograms represent three biological replicates for all RNA light-up aptamers in the presence and absence of the DFHBI-1T. Information regarding the construct, concentration of dye, replicate and cell count can be found in the tables above the histograms, with TB=Tornado Broccoli RNA aptamer, B=F30-2xdBroccoli, and S=tRNA-Spinach RNA aptamer.

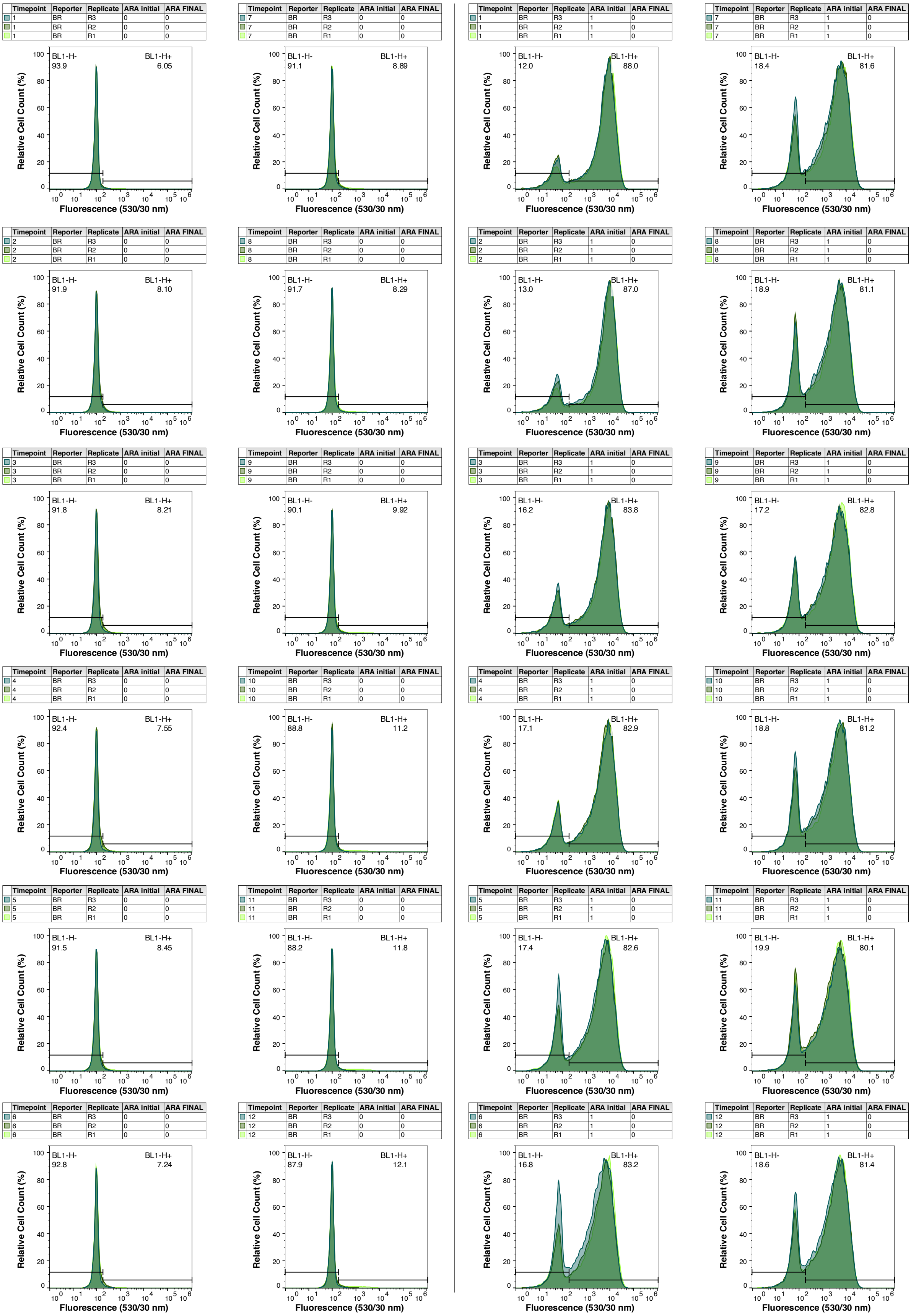

Supplementary Figure 10. Histograms for comparison of dynamics. Samples taken every 45 min for 12 timepoints for F30-2xdBroccoli 0%-0% L-arabinose (first two columns) and 1%-0% L-arabinose (last two columns). Cells were gated for the positive (BL1-H+) and negative (BL1-H-) populations to analyse the percentage of cells activated and inactivated, respectively. Gating indicated by the horizontal bars. 0%-0% sample remains off over time with ≈90% of cells within that gate. 1%-0% sample lose fluorescence signal over time whereas the negative population (BL1-H-) increases over time.

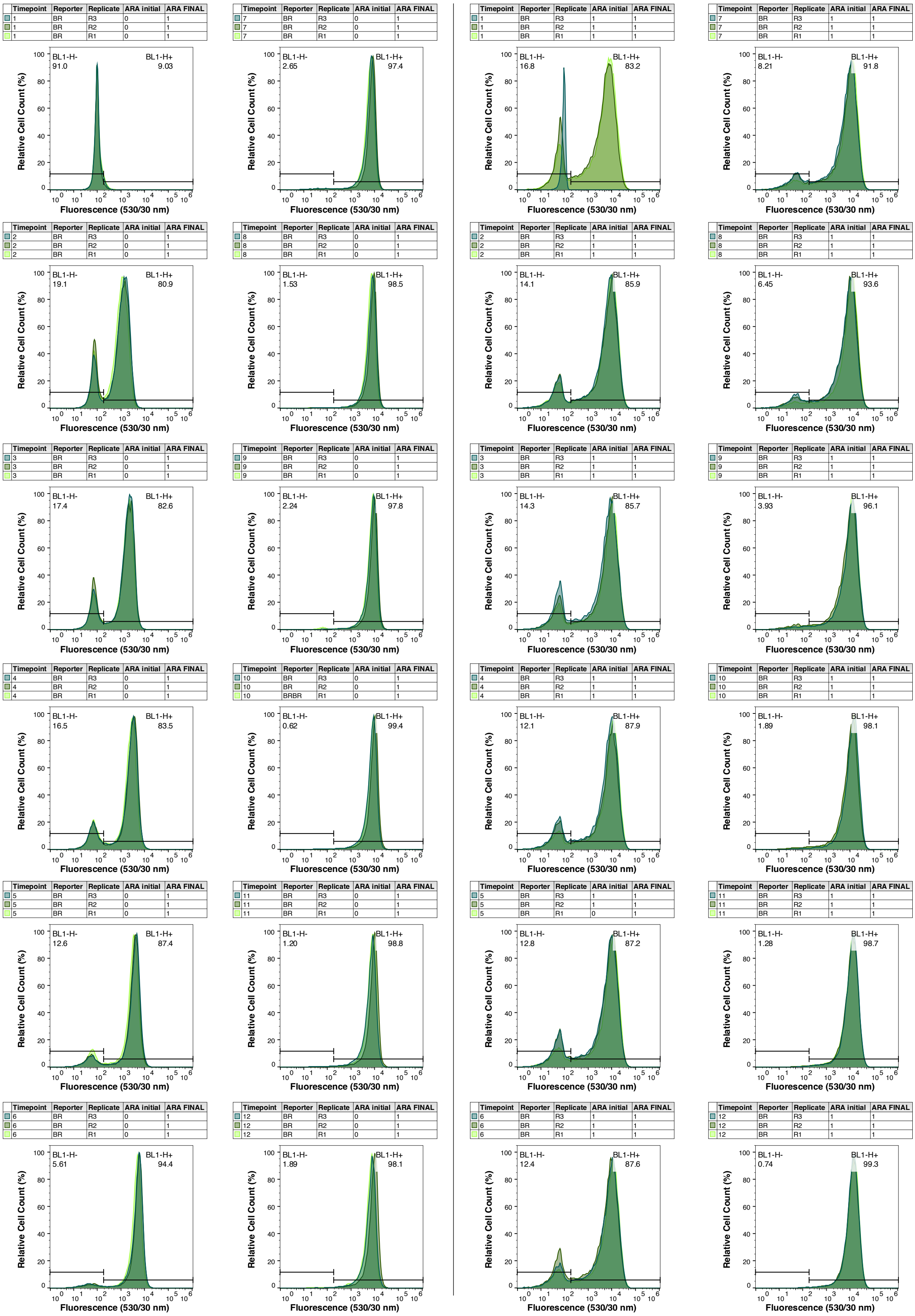

Supplementary Figure 11. **Histograms for comparison of dynamics.** Samples taken every 45 minutes for 12 timepoints for F30-2xdBroccoli 0%-1% L-arabinose (first two columns) and 1%-1% L-arabinose (last two columns). Cells were gated for the positive (BL1-H+) and negative (BL1-H-) populations to analyse the percentage of cells activated and inactivated, respectively. Gating indicated by the horizontal bars. The fluorescent signal of 0%-1% sample increases over time and the percentage of the positive population increase from 80% to 98% whereas the negative population decreases. The fluorescent signal of 1%-1% sample starts within the activated gate (BL1-H+) and increases over time.

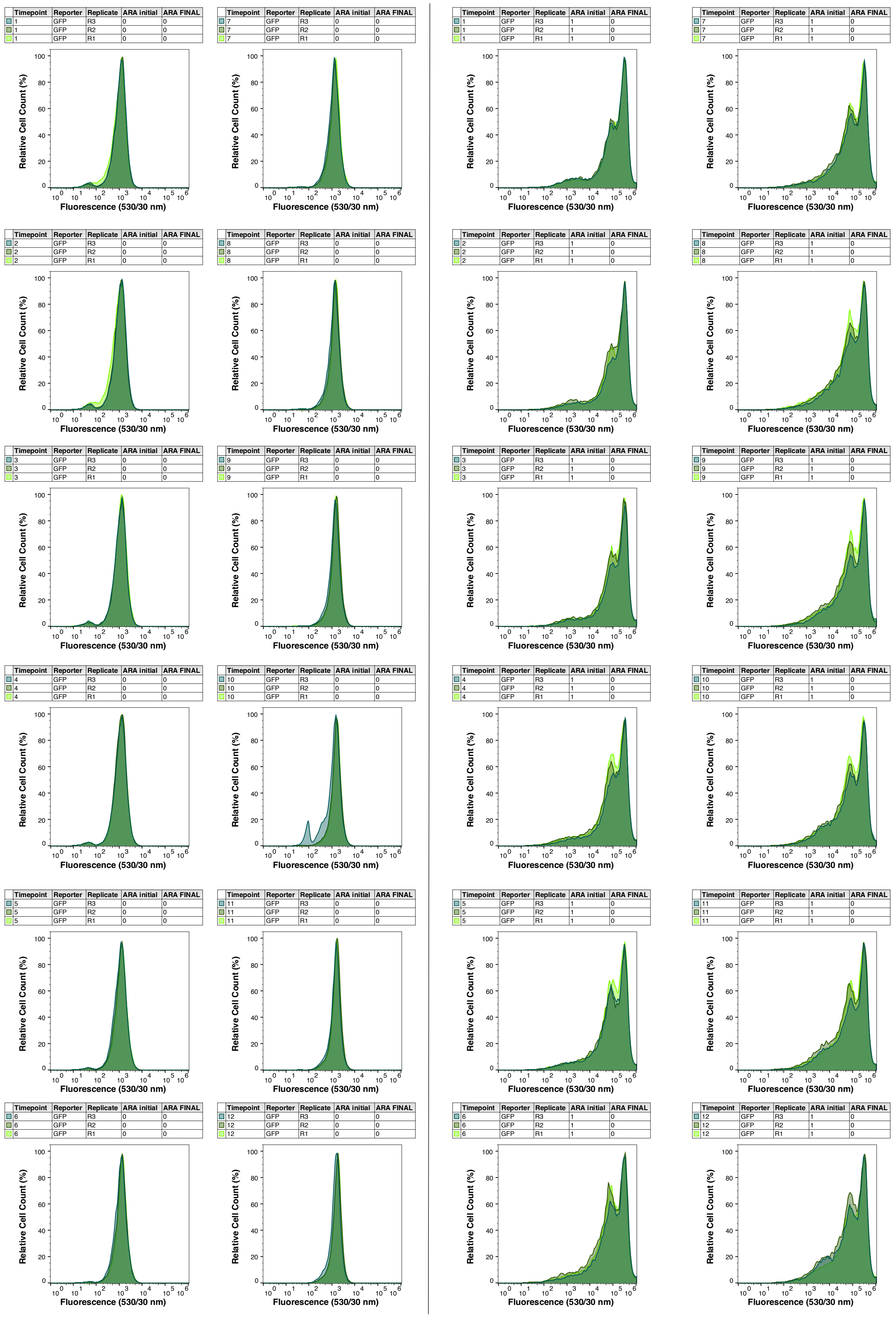

Supplementary Figure 12. **Histograms for comparison of dynamics.** Samples taken every 45 min for 12 timepoints for sfGFP 0%-0% L-arabinose (first two columns) and 1%-0% L-arabinose (last two columns). 0%-0% sample remains off over time with no increase in the fluorescence signal. The histogram for the 1%-0% sample changes over time as the fluorescent signal starts decreasing.

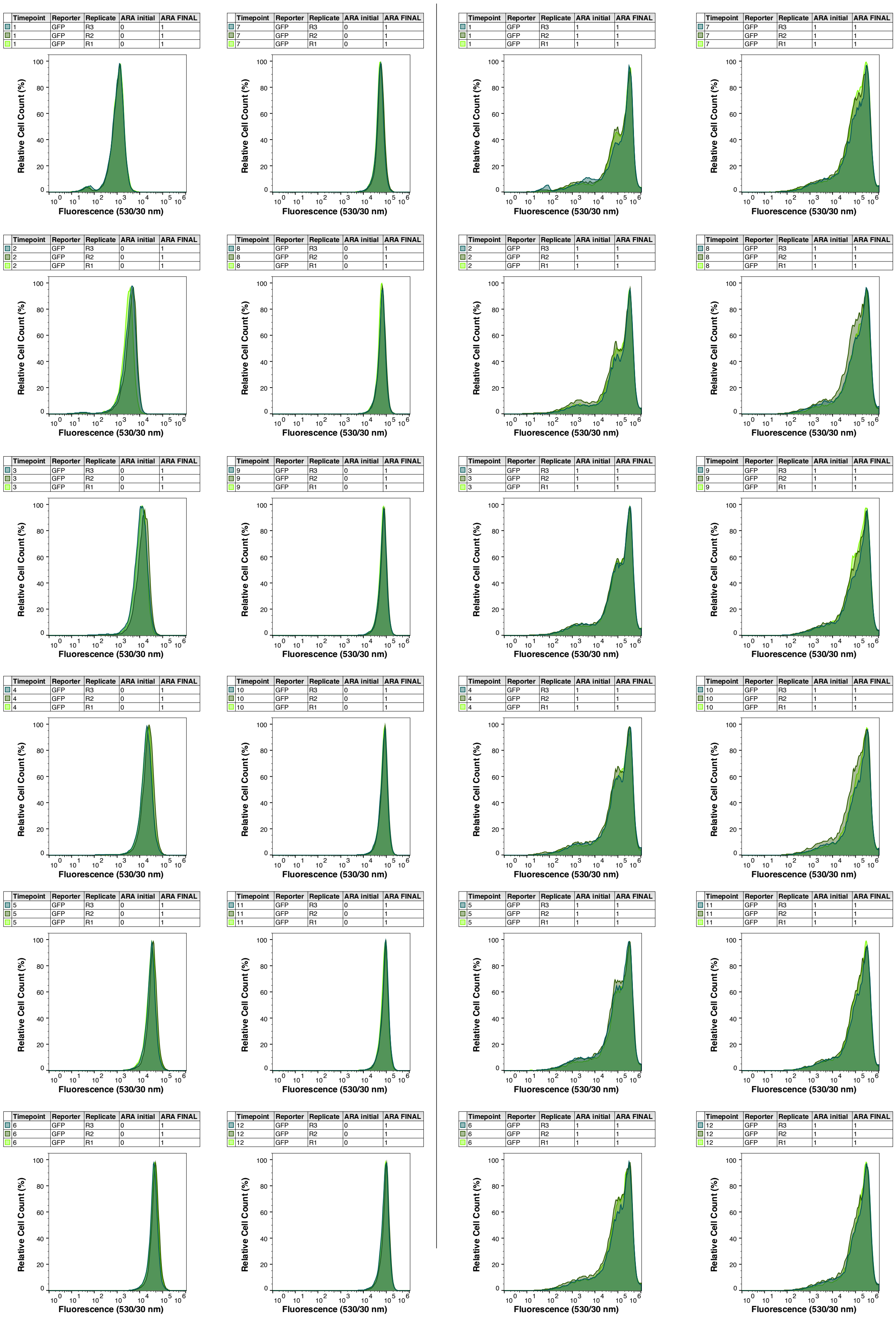

Supplementary Figure 13. **Histograms for comparison of dynamics.** Samples taken every 45 min for 12 timepoints for sfGFP 1%-0% L-arabinose (first two columns) and 1%-1% L-arabinose (last two columns). The fluorescent signal for the 0%-0% sample increases overtime whereas the activated sample 1%-1% is always activated.

Supplementary Table 1. P-values calculated with Tukey's multiple comparisons tests for the activation of the RNA light-up fluorescence signals (0 µM vs 160 µM of DFHBI-1T). Statistical analysis was performed using Graph Prism version 9.4.1 for three biological replicates. The samples with statistically significant activation are highlighted in green.

0 µM vs 160 µM F30-2xdBroccoli

		J23116	J23107	J23106	J23118	J23101	J23102	J23100	J23119
37ºC	Fructose	0.0012	<0.0001	0.0129	<0.0001	<0.0001	<0.0001	<0.0001	<0.0001
	Glucose	0.0300	<0.0001	0.0464	<0.0001	<0.0001	<0.0001	<0.0001	<0.0001
	Sucrose	0.6906	<0.0001	<0.0001	<0.0001	<0.0001	<0.0001	<0.0001	>0.9999
30ºC	Fructose	0.0115	0.5592	0.9091	0.9527	<0.0001	0.0002	0.6594	0.0144
	Glucose	>0.9999	<0.0001	0.9937	<0.0001	<0.0001	<0.0001	<0.0001	<0.0001
	Sucrose	>0.9999	0.9911	>0.9999	0.9970	0.0060	0.5965	>0.9999	>0.9999

0 µM vs 160 µM Tornado Broccoli

		J23116	J23107	J23106	J23118	J23101	J23102	J23100	J23119
37ºC	Fructose	0.8099	0.9894	0.0120	<0.0001	<0.0001	0.0003	0.0011	0.0008
	Glucose	>0.9999	0.9944	<0.0001	<0.0001	<0.0001	<0.0001	<0.0001	0.9989
	Sucrose	>0.9999	>0.9999	0.8448	0.9854	<0.0001	<0.0001	0.7812	0.1233
30ºC	Fructose	0.9993	0.9993	>0.9999	>0.9999	0.0134	0.9855	>0.9999	>0.9999
	Glucose	>0.9999	0.7958	0.9835	0.9775	0.0005	0.9961	>0.9999	0.9999
	Sucrose	>0.9999	>0.9999	0.9995	>0.9999	0.0488	0.9985	>0.9999	>0.9999

0 µM vs 160 µM tRNA-Spinach

		J23116	J23107	J23106	J23118	J23101	J23102	J23100	J23119
37ºC	Fructose	0.0028	0.4527	0.0904	<0.0001	<0.0001	<0.0001	<0.0001	<0.0001
	Glucose	0.9195	0.0004	0.0002	0.2552	0.0084	<0.0001	0.0002	<0.0001
	Sucrose	0.6831	>0.9999	0.9997	0.0090	0.7904	0.9537	0.02300	-0.0750
30ºC	Fructose	>0.9999	>0.9999	>0.9999	>0.9999	0.1306	0.0448	0.1207	<0.0001
	Glucose	0.5531	0.0388	0.0013	0.0002	0.0026	<0.0001	0.0267	<0.0001
	Sucrose	0.3293	0.0059	0.0147	0.0147	0.7860	0.2913	>0.9999	0.9007

Supplementary Table 2. P-values calculated with Tukey's multiple comparisons tests for F30-2xdBroccoli with the promoter library in the presence of 160 µM. Statistical analysis was performed using Graph Prism version 9.4.1 for three biological replicates and corresponds with the data displayed in Figure 1c. Samples with statistical differences are highlighted in green.

F30-2xdBroccoli M9 Fructose 30ºC

	J23116	J23107	J23106	J23118	J23101	J23102	J23100	J23119	
J23116		0.0621	0.4785	0.7139	0.0649	0.9801	0.6315	>0.9999	J23116
J23107	>0.9999		0.9989	0.9807	<0.0001	0.0014	0.9917	0.0693	J23107
J23106	0.9849	>0.9999		>0.999	<0.0001	0.0257	>0.9999	0.5092	J23106
J23118	0.0944	0.0005	0.0002		0.0002	0.0614	>0.9999	0.7543	J23118
J23101	<0.0001	<0.0001	<0.0001	<0.0001		0.7286	0.0001	0.0581	J23101
J23102	0.0006	<0.0001	<0.0001	>0.9999	<0.0001		0.0454	0.9738	J23102
J23100	0.3882	0.0030	0.0010	>0.9999	<0.0001	0.9479		0.6625	J23100
J23119	<0.0001	<0.0001	<0.0001	0.0049	0.0015	0.4786	0.0009		J23119
	J23116	J23107	J23106	J23118	J23101	J23102	J23100	J23119	

F30-2xdBroccoli M9 Fructose 37ºC

F30-2xdBroccoli M9 Glucose 30ºC

	J23116	J23107	J23106	J23118	J23101	J23102	J23100	J23119	
J23116		>0.9999	0.9491	<0.0001	<0.0001	<0.0001	<0.0001	0.2817	J23116
J23107	>0.9999		0.4905	<0.0001	<0.0001	<0.0001	<0.0001	0.8085	J23107
J23106	0.9939	0.9982		<0.0001	<0.0001	<0.0001	<0.0001	0.0067	J23106
J23118	<0.0001	<0.0001	<0.0001		<0.0001	0.0237	<0.0001	0.0009	J23118
J23101	<0.0001	<0.0001	<0.0001	0.0014		<0.0001	0.4114	<0.0001	J23101
J23102	<0.0001	<0.0001	<0.0001	0.1047	<0.0001		<0.0001	<0.0001	J23102
J23100	<0.0001	<0.0001	<0.0001	0.2569	0.7247	<0.0001		<0.0001	J23100
J23119	<0.0001	<0.0001	<0.0001	0.0017	<0.0001	0.9501	<0.0001		J23119
	J23116	J23107	J23106	J23118	J23101	J23102	J23100	J23119	

F30-2xdBroccoli M9 Glucose 37ºC

F30-2xdBroccoli M9 Sucrose 30ºC

	J23116	J23107	J23106	J23118	J23101	J23102	J23100	J23119	
J23116		>0.9999	>0.9999	>0.9999	0.0566	0.9022	>0.9999	>0.9999	J23116
J23107	0.3315		>0.9999	0.9862	0.0111	0.5281	>0.9999	0.9997	J23107
J23106	<0.0001	<0.0001		>0.9999	0.0670	0.9266	>0.9999	>0.9999	J23106
J23118	<0.0001	<0.0001	0.0133		0.2613	0.9992	>0.9999	>0.9999	J23118
J23101	<0.0001	<0.0001	<0.0001	<0.0001		0.8733	0.0556	0.1163	J23101
J23102	<0.0001	<0.0001	<0.0001	<0.0001	<0.0001		0.8994	0.9788	J23102
J23100	<0.0001	<0.0001	0.5869	0.8657	<0.0001	<0.0001		>0.9999	J23100
J23119	0.9857	0.0157	0.0010	<0.0001	<0.0001	<0.0001	<0.0001		J23119
	J23116	J23107	J23106	J23118	J23101	J23102	J23100	J23119	

F30-2xdBroccoli M9 Sucrose 37ºC

Supplementary Table 3. P-values calculated with Tukey's multiple comparisons tests for Tornado Broccoli with the promoter library in the presence of 160 µM. Statistical analysis was performed using Graph Prism version 9.4.1 for three biological replicates and corresponds with the data displayed in Figure 1c. Samples with statistical differences are highlighted in green.

Tornado Broccoli M9 Fructose 30ºC (upper right) / Tornado Broccoli M9 Fructose 37ºC (lower left)

	J23116	J23107	J23106	J23118	J23101	J23102	J23100	J23119	
**J23116**		0.9988	0.9986	>0.9999	0.0127	0.0653	0.8683	0.9208	**J23116**
**J23107**	>0.9999		>0.9999	>0.9999	0.1648	0.4988	>0.9999	>0.9999	**J23107**
**J23106**	0.4248	0.7514		>0.9999	0.1679	0.5050	>0.9999	>0.9999	**J23106**
**J23118**	<0.0001	0.0003	0.0816		0.0646	0.2542	0.9965	0.9990	**J23118**
**J23101**	<0.0001	<0.0001	<0.0001	<0.0001		>0.9999	0.5709	0.4803	**J23101**
**J23102**	0.0026	0.0103	0.6650	0.9951	<0.0001		0.9267	0.8765	**J23102**
**J23100**	<0.0001	<0.0001	0.0002	0.6575	0.0001	0.0795		>0.9999	**J23100**
**J23119**	<0.0001	<0.0001	0.0032	0.9936	<0.0001	0.4681	0.9997		**J23119**
	**J23116**	**J23107**	**J23106**	**J23118**	**J23101**	**J23102**	**J23100**	**J23119**	

Tornado Broccoli M9 Glucose 30ºC (upper right) / Tornado Broccoli M9 Glucose 37ºC (lower left)

	J23116	J23107	J23106	J23118	J23101	J23102	J23100	J23119	
**J23116**		>0.9999	>0.9999	0.8013	0.0369	0.0954	0.9999	>0.9999	**J23116**
**J23107**	0.0016		>0.9999	0.9111	0.0660	0.1599	>0.9999	>0.9999	**J23107**
**J23106**	0.3587	0.6253		0.9577	0.0965	0.2217	>0.9999	>0.9999	**J23106**
**J23118**	<0.0001	<0.0001	<0.0001		0.8903	0.9850	0.9985	0.6458	**J23118**
**J23101**	<0.0001	<0.0001	<0.0001	<0.0001		>0.9999	0.2504	0.0195	**J23101**
**J23102**	<0.0001	<0.0001	<0.0001	0.6598	<0.0001		0.4775	0.0533	**J23102**
**J23100**	<0.0001	<0.0001	<0.0001	<0.0001	<0.0001	<0.0001		0.9979	**J23100**
**J23119**	0.0079	>0.9999	0.9261	<0.0001	<0.0001	<0.0001	<0.0001		**J23119**
	**J23116**	**J23107**	**J23106**	**J23118**	**J23101**	**J23102**	**J23100**	**J23119**	

Tornado Broccoli M9 Sucrose 30ºC (upper right) / Tornado Broccoli M9 Sucrose 37ºC (lower left)

	J23116	J23107	J23106	J23118	J23101	J23102	J23100	J23119	
**J23116**		>0.9999	0.9944	>0.9999	0.1212	0.7230	>0.9999	>0.9999	**J23116**
**J23107**	<0.0001		>0.9999	>0.9999	0.3331	0.9550	>0.9999	>0.9999	**J23107**
**J23106**	<0.0001	0.9993		0.9993	0.7862	>0.9999	0.9989	0.9999	**J23106**
**J23118**	<0.0001	>0.9999	>0.9999		0.1896	0.8453	>0.9999	>0.9999	**J23118**
**J23101**	<0.0001	<0.0001	<0.0001	<0.0001		0.9977	0.1761	0.2459	**J23101**
**J23102**	<0.0001	<0.0001	<0.0001	<0.0001	<0.0001		0.8267	0.9039	**J23102**
**J23100**	<0.0001	0.9995	>0.9999	>0.9999	<0.0001	<0.0001		>0.9999	**J23100**
**J23119**	<0.0001	0.9368	>0.9999	0.9854	<0.0001	<0.0001	>0.9999		**J23119**
	**J23116**	**J23107**	**J23106**	**J23118**	**J23101**	**J23102**	**J23100**	**J23119**	

Supplementary Table 4. P-values calculated with Tukey's multiple comparisons tests for tRNA-Spinach with the promoter library in the presence of 160 µM. Statistical analysis was performed using Graph Prism version 9.4.1 for three biological replicates and corresponds with the data displayed in Figure 1c. Samples with statistical differences are highlighted in green.

tRNA-Spinach M9 Fructose 30ºC

	J23116	J23107	J23106	J23118	J23101	J23102	J23100	J23119	
**J23116**		0.9995	0.9999	>0.9999	0.1340	0.0409	0.3262	<0.0001	**J23116**
**J23107**	0.9991		>0.9999	0.9997	0.0120	0.0029	0.0397	<0.0001	**J23107**
**J23106**	0.9996	0.7772		>0.9999	0.0160	0.0040	0.0520	<0.0001	**J23106**
**J23118**	<0.0001	<0.0001	<0.0001		0.1207	0.0363	0.3001	<0.0001	**J23118**
**J23101**	<0.0001	<0.0001	<0.0001	0.9270		>0.9999	>0.9999	0.1448	**J23101**
**J23102**	<0.0001	<0.0001	<0.0001	>0.9999	0.8703		0.9995	0.3749	**J23102**
**J23100**	0.0007	<0.0001	0.0098	<0.0001	<0.0001	<0.0001		0.0505	**J23100**
**J23119**	<0.0001	<0.0001	<0.0001	<0.0001	<0.0001	<0.0001	<0.0001		**J23119**
	**J23116**	**J23107**	**J23106**	**J23118**	**J23101**	**J23102**	**J23100**	**J23119**	

tRNA-Spinach M9 Fructose 37ºC

tRNA-Spinach M9 Glucose 30ºC

	J23116	J23107	J23106	J23118	J23101	J23102	J23100	J23119	
**J23116**		0.9096	0.0687	0.5732	0.0040	<0.0001	0.0687	<0.0001	**J23116**
**J23107**	0.0067		0.0007	0.0168	<0.0001	<0.0001	0.0007	<0.0001	**J23107**
**J23106**	<0.0001	0.2811		0.9971	0.9983	0.0788	>0.9999	0.0065	**J23106**
**J23118**	0.9360	0.3086	0.0003		0.6135	0.0040	0.9971	0.0002	**J23118**
**J23101**	<0.0001	0.5399	>0.9999	0.0009		0.5732	0.9983	0.1031	**J23101**
**J23102**	<0.0001	<0.0001	<0.0001	<0.0001	<0.0001		0.0788	0.9995	**J23102**
**J23100**	<0.0001	0.0050	0.9195	<0.0001	0.7018	<0.0001		0.0065	**J23100**
**J23119**	<0.0001	<0.0001	<0.0001	<0.0001	<0.0001	0.0005	<0.0001		**J23119**
	**J23116**	**J23107**	**J23106**	**J23118**	**J23101**	**J23102**	**J23100**	**J23119**	

tRNA-Spinach M9 Glucose 37ºC

tRNA-Spinach M9 Sucrose 30ºC

	J23116	J23107	J23106	J23118	J23101	J23102	J23100	J23119	
**J23116**		>0.9999	>0.9999	0.5864	0.9994	0.1131	0.0006	<0.0001	**J23116**
**J23107**	0.8648		>0.9999	0.9518	>0.9999	0.4282	0.0043	<0.0001	**J23107**
**J23106**	<0.0001	<0.0001		0.9328	>0.9999	0.3842	0.0035	<0.0001	**J23106**
**J23118**	0.9391	>0.9999	<0.0001		0.9914	0.9996	0.2047	0.0013	**J23118**
**J23101**	0.0757	0.9467	<0.0001	0.8777		0.6188	0.0094	<0.0001	**J23101**
**J23102**	<0.0001	<0.0001	0.9537	<0.0001	0.0060		0.7721	0.0177	**J23102**
**J23100**	<0.0001	0.0005	0.6054	0.0003	0.0377	>0.9999		0.7721	**J23100**
**J23119**	0.0008	0.0986	0.0090	0.0617	0.9117	0.3284	0.7737		**J23119**
	**J23116**	**J23107**	**J23106**	**J23118**	**J23101**	**J23102**	**J23100**	**J23119**	

tRNA-Spinach M9 Sucrose 37ºC

Supplementary Table 5. Statistics and Tukey's multiple comparisons tests for the heterogeneity analysis of Tornado Broccoli RNA aptamer. Geometric Mean (G.Mean), Standard Deviation (SD), Coefficient of Variation (CV) and Cell Count for three biological replicates were calculated using FlowJo Software. Statistical analysis (multiple comparisons test) was performed using Graph Prism version 9.4.1.

	**Tornado Broccoli RNA aptamer**								
	**G. Mean (a.u.)**			**SD (a.u.)**			**CV (%)**		
	**R1**	**R2**	**R3**	**R1**	**R2**	**R3**	**R1**	**R2**	**R3**
**J23116**	102	109	106	22.3	25.3	23.4	21.3	22.5	21.6
**J23116 + DFHBI-1T**	99.5	107	104	26.8	28	26.4	26	25.2	24.7
**J23118**	95.9	96.5	97.7	24.3	24.1	23.1	24.5	24.1	23
**J23118 + DFHBI-1T**	425	444	403	391	419	369	70.2	71.7	70.1
**J23100**	101	101	103	32	34.7	34.9	30.4	32.7	32.4
**J23110 + DFHBI-1T**	688	748	849	861	877	903	80.4	77.1	72.4
**J23119**	101	102	102	28.6	29.3	28.4	27.2	27.7	26.7
**J23119 + DFHBI-1T**	418	748	408	521	716	527	82	68.1	83.8

**Tukey's multiple comparisons test**	**Mean 1**	**Mean 2**	**Mean Diff.**	**Below threshold**	**Adjusted P Value**
116TB vs. 116TB + dye	105.7	103.5	2.167	No	>0.9999
116TB vs. 118TB	105.7	96.7	8.967	No	>0.9999
116TB vs. 118TB + dye	105.7	424	-318.3	Yes	0.0017
116TB vs. 100TB	105.7	101.7	4	No	>0.9999
116TB vs. 100TB + dye	105.7	761.7	-656	Yes	<0.0001
116TB vs. 119TB	105.7	101.7	4	No	>0.9999
116TB vs. 119TB+dye	105.7	524.7	-419	Yes	<0.0001
116TB + dye vs. 118TB	103.5	96.7	6.8	No	>0.9999
116TB + dye vs. 118TB + dye	103.5	424	-320.5	Yes	0.0015
116TB + dye vs. 100TB	103.5	101.7	1.833	No	>0.9999
116TB + dye vs. 100TB + dye	103.5	761.7	-658.2	Yes	<0.0001
116TB + dye vs. 119TB	103.5	101.7	1.833	No	>0.9999
116TB + dye vs. 119TB+dye	103.5	524.7	-421.2	Yes	<0.0001
118TB vs. 118TB + dye	96.7	424	-327.3	Yes	0.0012
118TB vs. 100TB	96.7	101.7	-4.967	No	>0.9999
118TB vs. 100TB + dye	96.7	761.7	-665	Yes	<0.0001
118TB vs. 119TB	96.7	101.7	-4.967	No	>0.9999
118TB vs. 119TB+dye	96.7	524.7	-428	Yes	<0.0001
118TB + dye vs. 100TB	424	101.7	322.3	Yes	0.0015
118TB + dye vs. 100TB + dye	424	761.7	-337.7	Yes	0.0009
118TB + dye vs. 119TB	424	101.7	322.3	Yes	0.0015
118TB + dye vs. 119TB+dye	424	524.7	-100.7	No	0.7145
100TB vs. 100TB + dye	101.7	761.7	-660	Yes	<0.0001
100TB vs. 119TB	101.7	101.7	0	No	>0.9999
100TB vs. 119TB+dye	101.7	524.7	-423	Yes	<0.0001
100TB + dye vs. 119TB	761.7	101.7	660	Yes	<0.0001
100TB + dye vs. 119TB+dye	761.7	524.7	237	Yes	0.0221
119TB vs. 119TB+dye	101.7	524.7	-423	Yes	<0.0001

Supplementary Table 6. Statistics and Tukey's multiple comparisons tests for the heterogeneity analysis of F30-2xdBroccoli RNA aptamer. Geometric Mean, Mean, Mode and Cell Count for the three biological replicates were calculated using FlowJo Software. Statistical analysis (multiple comparisons test) was performed using Graph Prism version 9.4.1.

	F30-2xdBroccoli RNA aptamer								
	G. Mean (a.u.)			SD (a.u.)			CV (%)		
	R1	R2	R3	R1	R2	R3	R1	R2	R3
**J23116**	95.3	94.8	95.9	19.9	19.2	19.4	20.4	19.8	19.8
**J23116 + DFHBI-1T**	258	237	232	223	204	203	68.4	67.9	69.2
**J23118**	95.4	95.2	96.6	20.2	20.3	20.2	20.7	20.8	20.4
**J23118 + DFHBI-1T**	3909	3936	4002	2963	2886	2896	58.8	56.8	56.6
**J23100**	94.9	94.7	95.1	20.6	20.5	21.1	21.2	21.1	21.6
**J23100 + DFHBI-1T**	4573	4274	4808	3511	3683	3570	58.8	62.6	57.5
**J23119**	98.6	95.7	94.2	22.1	21.6	20.4	21.8	22	21.2
**J23119 + DFHBI-1T**	6604	6443	6318	6777	6632	7132	65.3	65.9	68.7

Tukey's multiple comparisons test	Mean 1	Mean 2	Mean Diff.	Below threshold	Adjusted P Value
116B vs. 116B + dye	95.33	242.3	-147	No	0.7134
116B vs. 118B	95.33	95.73	-0.4	No	>0.9999
116B vs. 118B + dye	95.33	3949	-3854	Yes	<0.0001
116B vs. 100B	95.33	94.9	0.4333	No	>0.9999
116B vs. 100B + dye	95.33	4552	-4456	Yes	<0.0001
116B vs. 119B	95.33	96.17	-0.8333	No	>0.9999
116B vs. 119B+dye	95.33	6455	-6360	Yes	<0.0001
116B + dye vs. 118B	242.3	95.73	146.6	No	0.7159
116B + dye vs. 118B + dye	242.3	3949	-3707	Yes	<0.0001
116B + dye vs. 100B	242.3	94.9	147.4	No	0.7106
116B + dye vs. 100B + dye	242.3	4552	-4309	Yes	<0.0001
116B + dye vs. 119B	242.3	96.17	146.2	No	0.7187
116B + dye vs. 119B+dye	242.3	6455	-6213	Yes	<0.0001
118B vs. 118B + dye	95.73	3949	-3853	Yes	<0.0001
118B vs. 100B	95.73	94.9	0.8333	No	>0.9999
118B vs. 100B + dye	95.73	4552	-4456	Yes	<0.0001
118B vs. 119B	95.73	96.17	-0.4333	No	>0.9999
118B vs. 119B+dye	95.73	6455	-6359	Yes	<0.0001
118B + dye vs. 100B	3949	94.9	3854	Yes	<0.0001
118B + dye vs. 100B + dye	3949	4552	-602.7	Yes	<0.0001
118B + dye vs. 119B	3949	96.17	3853	Yes	<0.0001
118B + dye vs. 119B+dye	3949	6455	-2506	Yes	<0.0001
100B vs. 100B + dye	94.9	4552	-4457	Yes	<0.0001
100B vs. 119B	94.9	96.17	-1.267	No	>0.9999
100B vs. 119B+dye	94.9	6455	-6360	Yes	<0.0001
100B + dye vs. 119B	4552	96.17	4456	Yes	<0.0001
100B + dye vs. 119B+dye	4552	6455	-1903	Yes	<0.0001
119B vs. 119B+dye	96.17	6455	-6359	Yes	<0.0001

Supplementary Table 7. Statistics and Tukey's multiple comparisons tests for the heterogeneity analysis of tRNA-Spinach RNA aptamer. Geometric Mean, Mean, Mode and Cell Count for the three biological replicates were calculated using FlowJo Software. Statistical analysis was performed using Graph Prism version 9.4.1.

	tRNA-Spinach RNA aptamer								
	G. Mean (a.u.)			SD (a.u.)			CV (%)		
	R1	R2	R3	R1	R2	R3	R1	R2	R3
**J23116**	117	117	114	29.5	28.7	28.9	24.4	23.9	24.5
**J23116 + DFHBI-1T**	380	426	457	430	455	469	80.1	76.4	74.6
**J23118**	131	130	129	37.4	35.7	34.6	27.3	26.5	25.8
**J23118 + DFHBI-1T**	136	135	134	55.2	55.4	55.8	37.9	38.2	38.9
**J23100**	96.1	95.6	96.7	21.6	22	21.7	21.9	22.4	21.8
**J23100 + DFHBI-1T**	138	116	115	51.8	57.6	50.1	35.5	45.9	40.8
**J23119**	104	104	104	24.3	25.8	25.8	22.7	24.1	24.1
**J23119 + DFHBI-1T**	897	985	826	675	700	628	58	55.9	58.8

Tukey's multiple comparisons test	Mean 1	Mean 2	Mean Diff.	Below threshold	Adjusted P Value
116S vs. 116S + dye	116	135	-19	No	0.9943
116S vs. 118S	116	130	-14	No	0.9991
116S vs. 118S + dye	116	123	-7	No	>0.9999
116S vs. 100S	116	96.13	19.87	No	0.9926
116S vs. 100S + dye	116	421	-305	Yes	<0.0001
116S vs. 119S	116	104	12	No	0.9997
116S vs. 119S + dye	116	902.7	-786.7	Yes	<0.0001
116S + dye vs. 118S	135	130	5	No	>0.9999
116S + dye vs. 118S + dye	135	123	12	No	0.9997
116S + dye vs. 100S	135	96.13	38.87	No	0.7952
116S + dye vs. 100S + dye	135	421	-286	Yes	<0.0001
116S + dye vs. 119S	135	104	31	No	0.9208
116S + dye vs. 119S + dye	135	902.7	-767.7	Yes	<0.0001
118S vs. 118S + dye	130	123	7	No	>0.9999
118S vs. 100S	130	96.13	33.87	No	0.8823
118S vs. 100S + dye	130	421	-291	Yes	<0.0001
118S vs. 119S	130	104	26	No	0.9668
118S vs. 119S + dye	130	902.7	-772.7	Yes	<0.0001
118S + dye vs. 100S	123	96.13	26.87	No	0.9607
118S + dye vs. 100S + dye	123	421	-298	Yes	<0.0001
118S + dye vs. 119S	123	104	19	No	0.9943
118S + dye vs. 119S + dye	123	902.7	-779.7	Yes	<0.0001
100S vs. 100S + dye	96.13	421	-324.9	Yes	<0.0001
100S vs. 119S	96.13	104	-7.867	No	>0.9999
100S vs. 119S + dye	96.13	902.7	-806.5	Yes	<0.0001
100S + dye vs. 119S	421	104	317	Yes	<0.0001
100S + dye vs. 119S + dye	421	902.7	-481.7	Yes	<0.0001
119S vs. 119S + dye	104	902.7	-798.7	Yes	<0.0001

Supplementary Table 8. Statistics for sfGFP controls. Geometric Mean (G.mean), standard deviation (SD), and coefficient of variation (CV) were calculated using FlowJo Software for samples expressing complete and truncated sfGFP.

	sfGFP complete			sfGFP truncated		
	G.mean (a.u.)	SD (a.u.)	CV (%)	G.mean (a.u.)	SD (a.u.)	CV (%)
**J23116**	1425	512	33.6	94.6	19.6	20.3
**J23118**	25002	10604	38.8	97.5	22.5	22.4
**J23100**	126224	43695	32.6	126	46.1	34.3
**J23119**	271039	141038	45.8	96.3	20.5	20.8

Supplementary Table 9. Statistics to study protein and RNA aptamer dynamics. Geometric Mean and standard deviation across three biological replicates were calculated using FlowJo Software.

	Broccoli aptamer (1% - 1%)		Broccoli aptamer (1% - 0%)		Broccoli aptamer (0% - 1%)		Broccoli aptamer (0% - 0%)	
**Hours**	**G. Mean**	**SD**	**G. Mean**	**SD**	**G. Mean**	**SD**	**G. Mean**	**SD**
**0.0**	3710.333	2066.949	6001	403.2158	275	38	589.3333	471.6994
**0.8**	6349.333	243.4345	6232.667	344.0441	1150.333	98.35819	342.6667	118.0692
**1.5**	6418.667	472.9338	5391.667	225.5002	1871.667	61.82502	377	126.4397
**2.3**	6777.667	525.8444	4964.667	134.0012	2572.333	195.8579	393.6667	143.6431
**3.1**	6576	542.7126	4227	260.1788	3320.333	300.6499	413.3333	177.2465
**3.8**	6680.333	542.1626	3935.333	487.1923	4598	403.8576	415	106.3532
**4.6**	7194.667	567.5001	3662.667	241.1168	6094	636.554	421	192.281
**5.4**	7231.667	701.3803	3359.667	187.6761	6700	625.1984	495	207.3716
**6.2**	8315.333	534.515	3444.667	218.6717	7263.333	650.2894	502.6667	271.4725
**6.9**	9767.667	761.0863	3255.333	166.5723	7380.333	693.076	584.3333	391.0375
**7.6**	10292.67	236.3564	3073	177.0339	6880.333	880.0326	745	381.9332

	sfGFP protein (1% - 1%)		sfGFP protein (1% - 0%)		sfGFP protein (0% - 1%)		sfGFP protein (0% - 0%)	
**Hours**	**G. Mean**	**SD**	**G. Mean**	**SD**	**G. Mean**	**SD**	**G. Mean**	**SD**
**0.0**	85583	6105.159	90015.33	3130.822	1097.333	22.47962	1066.667	59.60145
**0.8**	82174.67	9552.882	95497.67	7968.186	3342.333	479.7607	1031.667	73.63649
**1.5**	81611	3861.099	90148.33	4144.678	11069.67	1450.466	1130.667	25.38372
**2.3**	77638.33	3706.281	81924	8116.832	19363.33	1874.311	1135.667	18.58315
**3.1**	79597.67	5919.428	79984	4776.516	30208.67	2822.924	1157.333	56.07436
**3.8**	79282.67	5857.822	72412.67	7625.311	40298	3090.91	1197	73.73602
**4.6**	81817	4187.425	78042	6126.502	50534.67	2439.544	1249	76.73982
**5.4**	88511	8935.403	67957	5564.231	58613.67	1798.488	1299	96.00521
**6.2**	92111.67	3938.213	70193.67	6736.482	66940.67	2423.578	1368.333	89.69021
**6.9**	95337.67	11481.74	67898.67	4385.931	71845.33	2342.117	1394.667	173.3272
**7.6**	100845	2902.358	66545.33	5368.533	78840.67	2763.193	1454.667	88.29685

Supplementary Table 10. Normalized Cell Count for F30-2xdBroccoli RNA light-up aptamer. Geometric Mean and standard deviation for the number of cells in the activated and inactivated populations across three biological replicates were calculated using FlowJo Software for each sample.

	Number of Cells in the activated gate							
	Broccoli aptamer (1% - 1%)		Broccoli aptamer (1% - 0%)		Broccoli aptamer (0% - 1%)		Broccoli aptamer (0% - 0%)	
**Hours**	**Mean**	**SD**	**Mean**	**SD**	**Mean**	**SD**	**Mean**	**SD**
**0.0**	53.60	45.84	84.10	1.31	6.70	2.30	3.69	2.05
**0.8**	82.77	0.68	83.27	1.05	80.03	2.06	6.16	1.79
**1.5**	81.23	1.72	81.90	0.26	81.53	1.76	6.13	2.35
**2.3**	82.63	1.45	81.97	0.55	83.53	0.21	5.61	2.31
**3.1**	82.03	1.62	80.23	1.53	87.73	0.80	5.58	2.55
**3.8**	82.90	1.87	80.07	2.86	91.37	0.76	5.88	1.52
**4.6**	86.57	1.53	80.13	1.36	93.63	0.74	5.87	2.75
**5.4**	88.43	1.38	79.57	0.75	94.47	0.45	5.32	2.58
**6.2**	91.87	0.71	81.17	0.72	94.93	0.29	5.81	3.61
**6.9**	94.23	0.90	79.67	1.11	96.00	0.70	6.37	4.18
**7.6**	94.83	0.31	79.07	1.29	95.10	0.70	7.05	4.16

	Number of Cells in the non-activated gate							
	Broccoli aptamer (1% - 1%)		Broccoli aptamer (1% - 0%)		Broccoli aptamer (0% - 1%)		Broccoli aptamer (0% - 0%)	
**Hours**	**Mean**	**SD**	**Mean**	**SD**	**Mean**	**SD**	**Mean**	**SD**
**0.0**	45.37	46.75	13.50	1.61	93.30	2.35	96.13	2.31
**0.8**	14.43	0.97	14.10	1.45	19.90	2.12	93.83	1.78
**1.5**	15.97	2.31	16.07	0.46	18.37	1.76	93.83	2.38
**2.3**	14.07	2.14	16.30	0.50	16.17	0.25	94.30	2.33
**3.1**	14.93	2.19	18.60	1.80	11.43	0.97	94.37	2.65
**3.8**	14.00	2.43	18.87	3.10	5.97	0.43	94.03	1.56
**4.6**	9.97	1.81	18.90	1.39	2.61	0.56	94.07	2.80
**5.4**	8.04	1.68	19.67	0.90	1.72	0.22	94.63	2.65
**6.2**	4.05	0.79	18.03	0.81	1.46	0.63	94.13	3.63
**6.9**	1.91	0.73	19.67	1.16	0.98	0.50	93.53	4.36
**7.6**	1.13	0.16	20.37	1.34	1.35	0.57	92.80	4.30

Supplementary Table 11. Sequences used in this work.

Name	Part	Sequence (5’-3’)	Ref
J23100	Prom.	ttgacggctagctcagtcctaggtacagtgctagc	(1,2)
J23116	Prom.	ttgacagctagctcagtcctagggactatgctagc	(1,2)
J23118	Prom.	ttgacggctagctcagtcctaggtattgtgctagc	(1,2)
J23119	Prom.	ttgacagctagctcagtcctaggtataatgctagc	(1,2)
J23106	Prom.	tttacggctagctcagtcctaggtatagtgctagc	(1,2)
J23107	Prom.	tttacggctagctcagccctaggtattatgctagc	(1,2)
J23101	Prom.	tttacagctagctcagtcctaggtattatgctagc	(1,2)
J23102	Prom	ttgacagctagctcagtcctaggtactgtgctagc	(1,2)
AraC-pBAD	Prom +CDS	ttatgacaacttgacggctacatcattcactttttcttcacaaccggcacggaactcgctcgggctggccc<br>cggtgcatttttaaatacccgcgagaaatagagttgatcgtcaaaaccaacattgcgaccgacggtgg<br>cgataggcatccgggtggtgctcaaaagcagcttcgcctggctgatacgttggtcctcgcgccagcttaa<br>gacgctaatccctaactgctggcggaaaagatgtgacagacgcgacggcgacaagcaaacatgctgtg<br>cgacgctggcgatatcaaaattgctgtctgccaggtgatcgctgatgtactgacaagcctcgcgtacccg<br>attatccatcggtggatggagcgactcgttaatcgcttccatgcgccgcagtaacaattgctcaagcaga<br>tttatcgccagcagctccgaatagcgcccttccccttgcccggcgttaatgatttgcccaaacaggtcgct<br>gaaatgcggctggtgcgcttcatccgggcgaaagaaccccgtattggcaaatattgacggccagttaag<br>ccattcatgccagtaggcgcgcggacgaaagtaaacccactggtgataccattcgcgagcctccggatg<br>acgaccgtagtgatgaatctctcctggcgggaacagcaaaatatcacccggtcggcaaacaaattctcg<br>tccctgatttttcaccaccccctgaccgcgaatggtgagattgagaatataacctttcattcccagcggtc<br>ggtcgataaaaaaatcgagataaccgttggcctcaatcggcgttaaacccgccaccagatgggcatta<br>aacgagtatcccggcagcaggggatcattttgcgcttcagccatacttttcatactcccgccattcagag<br>aagaaaccaattgtccatattgcatcagacattgccgtcactgcgtcttttactggctcttctcgctaacc<br>aaaccggtaaccccgcttattaaaagcattctgtaacaaagcgggaccaaagccatgacaaaaacgcg<br>taacaaaagtgtctataatcacggcagaaaagtccacattgattatttgcacggcgtcacactttgctat<br>gccatagcatttttatccataagattagcggatcctacctgacgctttttatcgcaactctctactgtttctc<br>catacccgtttttttgggctagc	(3)
BBa B0034	RBS	aaagaggagaaatactag	(2)
F30-2xd Broccoli	RNA aptamer	ttgccatgtgtatgtgggagacggtcgggtccatctgagacggtcgggtccagatattcgtatctgtcga<br>gtagagtgtgggctcagatgtcgagtagagtgtgggctcccacatactctgatgatccagacggtcggg<br>tccatctgagacggtcgggtccagatattcgtatctgtcgagtagagtgtgggctcagatgtcgagtaga<br>gtgtgggctggatcattcatggcaa	(4)
Tornado Broccoli	RNA aptamer	gccatcagtcgccggtcccaagcccggataaaatgggagggggcgggaaaccgcctaaccatgccga<br>ctgatggcagttgccatgtgtatgtgggagacggtcgggtccatctgagacggtcgggtccagatattcg<br>tatctgtcgagtagagtgtgggctcagatgtcgagtagagtgtgggctcccacatactctgatgatccag<br>acggtcgggtccatctgagacggtcgggtccagatattcgtatctgtcgagtagagtgtgggctcagatg<br>tcgagtagagtgtgggctggatcattcatggcaactgccatcagtcggcgtggactgtagaacactgcc<br>aatgccggtcccaagcccggataaaagtggagggtacagtccacgc	(5)
tRNA-Spinach	RNA aptamer	gcccggatagctcagtcggtagagcagcggccggacgcaactgaatgaaatggtgaaggacgggtcc<br>aggtgtggctgcttcggcagtgcagcttgttgagtagagtgtgagctccgtaactagtcgcgtccggccg<br>cgggtccagggttcaagtccctgttcgggcgcca	(6,7)
sfGFP	CDS	atgcgtaaaggcgaggagctgttcactggtgtcgtccctattctggtggaactggatggtgatgtcaacg<br>gtcataagttttccgtgcgtggcgagggtgaaggtgacgcaactaatggtaaactgacgctgaagttca<br>tctgtactactggtaaactgccggtaccttggccgactctggtaacgacgctgacttatggtgttcagtgc<br>tttgctcgttatccggaccatatgaagcagcatgacttcttcaagtccgccatgccggaaggctatgtgc<br>aggaacgcacgatttcctttaaggatgacggcacgtacaaaacgcgtgcggaagtgaaatttgaaggc<br>gataccctggtaaaccgcattgagctgaaaggcattgactttaaagaagacggcaatatcctgggccat<br>aagctggaatacaattttaacagccacaatgtttacatcaccgccgataaacaaaaaaatggcattaa	(8)

		agcgaattttaaaattcgccacaacgtggaggatggcagcgtgcagctggctgatcactaccagcaaa acactccaatcggtgatggtcctgttctgctgccagacaatcactatctgagcacgcaaagcgttctgtct aaagatccgaacgagaaacgcgatcatatggttctgctggagttcgtaaccgcagcgggcatcacgca tggtatggatgaactgtacaaa	

Supplementary Table 12. Primers used in in RT-qPCR experiments.

Name	Exp	Sequence (5'-3')	Tm	Product size
16S Forward	qPCR	GCAGCAGTGGGGAATATTGC	66.6	200bp
16S Reverse	qPCR	CGCTTGCACCCTCCGTATTA	66.9	
Broccoli Forward	qPCR	CGGTCGGGTCCAGATATTCGTATC	70	190bp
Broccoli Reverse	qPCR	CATGAATGATCCAGCCCACACTCTA	70	
Tornado Forward	qPCR	AACCATGCCGACTGATGGCAGGAGA	77	96bp
Tornado Reverse	qPCR	CTACAGTCCACGCCGACTGATGGCA	77	
Spinach Forward	qPCR	CCCGGATAGCTCAGTCGGTA	77	96bp
Spinach Reverse	qPCR	CCGAACAGGGACTTGAACC	77	

References

1. Anderson JC, Dueber JE, Leguia M, Wu GC, Arkin AP, Keasling JD. BglBricks: A flexible standard for biological part assembly. J Biol Eng. 2010;4:1–12.
2. Anderson promoter collection. Available: http://parts.igem.org/Promoters/Catalog/Anderson.
3. Ceroni F, Algar R, Stan GB, Ellis T. Quantifying cellular capacity identifies gene expression designs with reduced burden. Nat Methods. 2015;12(5):415–8.
4. Filonov GS, Kam CW, Song W, Jaffrey SR. In-gel imaging of RNA processing using broccoli reveals optimal aptamer expression strategies. Chem Biol. 2015;22(5):649–60.
5. Litke JL, Jaffrey SR. Highly efficient expression of circular RNA aptamers in cells using autocatalytic transcripts. Nat Biotechnol. 2019;37(6):667–75.
6. Song W, Strack RL, Svensen N, Ja SR. Plug-and-Play Fluorophores Extend the Spectral Properties of Spinach. J Am Chem Soc. 2014;136(4):1198–201.
7. Pothoulakis G, Ceroni F, Reeve B, Ellis T. The Spinach RNA Aptamer as a Characterization Tool for Synthetic Biology. ACS Synth Biol. 2014;3(3):182–7.
8. Pédelacq JD, Cabantous S, Tran T, Terwilliger TC, Waldo GS. Engineering and characterization of a superfolder green fluorescent protein. Nat Biotechnol. 2006;24(1):79–88.
